# Integrative Population Genomics Reveals Niche Differentiation and Gene Flow in Chinese Sclerophyllous Oaks (*Quercus* Sect. *Ilex*)

**DOI:** 10.3390/plants14152403

**Published:** 2025-08-03

**Authors:** Miao-Miao Ju, Ming Yue, Gui-Fang Zhao

**Affiliations:** 1 Xi’an Botanical Garden of Shaanxi Province (Institute of Botany of Shaanxi Province), Shaanxi Academy of Science, Xi’an 710061, China; jumm089@163.com; 2 Key Laboratory of Resource Biology and Biotechnology in Western China, Ministry of Education, College of Life Sciences, Northwest University, Xi’an 710069, China

**Keywords:** sclerophyllous oaks, reticulate evolution, gene flow, introgression, niche differentiation

## Abstract

Elucidating the coexistence mechanisms of rapidly diverging species has long been a challenge in evolutionary biology. Genome-wide polymorphic loci are expected to provide insights into the speciation processes of these closely related species. This study focused on seven Chinese sclerophyllous oaks, represented by *Quercus spinosa*, *Quercus aquifolioides*, *Quercus rehderiana*, *Quercus guyavifolia*, *Quercus monimotricha*, *Quercus semecarpifolia*, and *Quercus senescens*, employing 27,592 single-nucleotide polymorphisms to examine their phylogenetic relationships at the genomic level. Combined with genetic structure analysis, phylogenetic trees revealed that the genetic clustering of individuals was influenced by both geographic distance and ancestral genetic components. Furthermore, this study confirmed the existence of reticulate evolutionary relationships among the species. Frequent gene flow and introgression within the seven species were primarily responsible for the ambiguous interspecies boundaries, with hybridization serving as a major driver of reticulate evolution. Additionally, the seven species exhibited distinct differences in niche occupancy. By reconstructing the climatic adaptability of ancestral taxonomic units, we found that the climatic tolerance of each species displayed differential responses to 19 climatic factors. Consequently, ecological niche differentiation and variations in habitat adaptation contributed to the preservation of species boundaries. This study provides a comprehensive understanding of the speciation processes in rapidly diverging genera and underscores the significance of both genetic and ecological factors in the formation and maintenance of species boundaries.

## 1. Introduction

The genus *Quercus* (Fagaceae), encompassing ecologically and economically significant oak trees, exhibits a near-cosmopolitan distribution across Asia, Africa, Europe, and the Americas (Flora of China). Serving as a critical germplasm resource for Northern Hemisphere broad-leaved forests, it provides essential ecosystem services such as habitat stabilization and ecological restoration [1]. Through global-scale analyses utilizing a comprehensive dataset of distribution and functional traits for 407 species, the biogeographic patterns of the genus *Quercus* have been characterized [2]. The two primary *Quercus* diversity centers are in East Asia, particularly southern China, and in Central and southern Mexico. These regions exhibit high species richness, endemism, and adaptive radiation. Specifically, sclerophyllous oaks are concentrated in Southwest China, with disjunct populations in East Asian high mountains and Mediterranean-type ecosystems [3].

Recent advancements in phylogenomic methodologies and high-throughput sequencing are profoundly reshaping our understanding of oak evolution. Whole-genome sequencing-derived single-nucleotide polymorphism (SNP) datasets have successfully resolved taxonomic ambiguities among four ecologically dominant European white oaks (*Quercus petraea*, *Quercus robur*, *Quercus pubescens*, and *Quercus pyrenaica*), thereby establishing a robust molecular framework [4]. Building upon this foundation, Reutimann et al. [5] integrated diagnostic SNPs with geospatial analytics, demonstrating that microtopographic gradients and edaphic heterogeneity govern asymmetric introgression within hybrid zones of Swiss *Q. petraea* and *Q. pubescens*. Despite extensive gene flow observed within syngameon networks, *Quercus* species effectively maintain reproductive isolation through context-dependent barriers. While introgression filters in European white oaks (sect. *Quercus*) are well-characterized [6], the extent to which these mechanisms generalize across other oak lineages remains an unresolved question.

North American *Quercus* species exhibit complex phylogeographic patterns. Phylogenetic analyses based on RAD-seq data for 250 transcontinental species revealed a multi-radiation origin for North American lineages, demonstrating limited phylogenetic affinity to Mexican clades while retaining ancestral Eurasian connections. Consequently, the taxonomic framework for 62 species within this radiation was revised and reorganized [7]. In Mexico, ecological niche integrated phylogenomics applied to the hyperdiverse *Quercus laeta* complex underscores the necessity of cross-disciplinary approaches for precise species delimitation [8]. Concurrent genomic advancements within the red oak lineage include the generation of the first chromosome-scale genome assembly for *Quercus rubra* [9], achieved via PacBio-HiC synteny mapping. This approach elucidates the genomic constraints influencing diversification within North America’s section *Lobatae*. Collectively, evidence from these extensive continental-scale studies converges on a unified paradigm: *Quercus* diversification stems from the interplay between genomic conservatism, characterized by retained ancestral architectures, and ecological lability, driven by niche plasticity.

As the world’s second-largest global distribution center for oaks, China hosts exceptional *Quercus* diversity. Prior research on Chinese oaks have predominantly focused on functional traits and morphological characteristics, encompassing karyotype analysis and pollen morphology [10]. Recent syntheses have quantified covariation among functional traits of dominant *Quercus* species across their core distribution zones, elucidating climate-response mechanisms using multivariate statistical frameworks [11]. Sophisticated analytical approaches have further clarified the ecological drivers of species distributions. Multivariate analyses integrating climate indices with occurrence data have resolved niche partitioning among sclerophyllous oak congeners, identifying temperature seasonality and precipitation thresholds as critical determinants of both latitudinal ranges and elevational zonation [12]. Moreover, macroecological assessments testing the water–energy dynamics hypothesis against Asian oak diversity patterns have demonstrated that energy availability, particularly growing-season warmth, primarily governs spatial distribution patterns across subgenera, while hydrological factors modulate fine-scale assemblage composition [13].

Genomic research has not only provided high-resolution data for individual species, but has also enabled the investigation of broader evolutionary forces. For instance, genotype–environment association analyses previously identified climate-associated SNPs across diverse genes and functional categories in *Quercus acutissima* [14]. Additionally, a chromosome-scale genome assembly for *Quercus mongolica* was assembled through high-throughput sequencing, providing a more comprehensive reference genome for studying the genetic variation patterns in deciduous oaks [15]. Furthermore, extensive research has explored the impact of linked selection on genetic variation patterns within the subgenus *Cerris* and subgenus *Quercus* [16,17]. This research on foundational evolutionary processes complements studies on specific species like *Quercus variabilis*, *Q. mongolica*, and *Quercus liaotungensis* [18,19], which have detailed morphological characteristics and genetic diversity levels.

Synthesizing the current research, studies on the genetic diversity of *Quercus* in China have predominantly focused on deciduous oaks. Due to limitations in marker number and sample size, the exploration of genetic diversity patterns within sclerophyllous oaks remains incomplete. For instance, patterns of whole-genome variation both within and between sclerophyllous oaks are still unclear, and the extent and directionality of gene flow remain unexplored. Furthermore, the correlation between niche differentiation (e.g., differences in temperature, seasonality, and precipitation) and genomic divergence is unresolved. Therefore, to address these knowledge gaps regarding the genetic architecture and evolutionary background of *Quercus* in China, it is imperative to employ advanced genomic technologies, focusing on in-depth investigation of the understudied sclerophyllous oaks.

The southwestern region of China is widely recognized as the primary center of origin for sclerophyllous oaks [20]. Characterized by their leathery, hard leaves with sharply serrated margins, these taxa exhibit a remarkable adaptation to cold, seasonally arid, and hot climates. They are prevalent in tropical and subtropical alpine forests, where they frequently assume dominant or foundational roles, playing crucial ecological functions within their local environments [21]. A global phylogenetic framework for the genus *Quercus*, reconstructed using fossil data and restriction-site-associated DNA sequencing (RAD-seq), indicates that sclerophyllous oaks growing in the southwestern region of China mainly correspond to the Himalayan subalpine species of the *Quercus* sect. *Ilex* [22]. Previous geological, phylogeographic [23,24], and hybridization [25] studies focusing on sclerophyllous oaks have demonstrated the profound influence of the Himalaya–Hengduan Mountains’ uplift on the distribution patterns of these species. Based on the records in the Flora of China, we selected seven *Quercus* sect. *Ilex* species that grow in China as representatives of sclerophyllous oaks for this study, including *Quercus spinosa*, *Quercus aquifolioides*, *Quercus rehderiana*, *Quercus guyavifolia*, *Quercus monimotricha*, *Quercus semecarpifolia*, and *Quercus senescens*. These species frequently occur sympatrically or in adjacent areas within Southwest China, reflecting the high species diversity and complex phylogenetic relationships characteristic of this region. Investigating the genetic diversity distribution patterns within these seven species will provide valuable insights into the genetic variation and species boundary maintenance mechanisms of sclerophyllous oaks in China, thereby facilitating the comprehensive utilization of the nation’s abundant *Quercus* germplasm resources.

This study adopted a systematic approach to investigate and collect samples from these species across China. Using specific-locus amplified fragment sequencing (SLAF-Seq), a substantial amount of genome-wide SNP data was generated [26]. By integrating genomic and ecological data, we thoroughly investigated the evolutionary history and adaptive mechanisms of these species. This integrative approach aims to uncover the spatiotemporal processes underlying the formation of their diversity patterns and elucidate how ecological factors shape their evolutionary trajectories at the genomic level. Understanding the genetic variation patterns, population dynamics, and evolutionary mechanisms of sclerophyllous oaks in China is crucial for conserving biodiversity and maintaining ecosystem functioning.

## 2. Results

### 2.1. Identification and Development of SNP Sites

Through the application of SLAF-seq technology, a comprehensive dataset totaling 637.10 GB was generated from the library of all individuals. The proportion of raw sequence data with a quality score of Q30 in the 291 individuals ranged from 87.71% to 94.36%, with an average of 91.52%. The GC content varied between 38.15% and 43.25%, with an average of 40.75%. From the samples, a total of 84,421 to 245,587 base pairs (bp) of SLAF-seq data were produced, with an average sequencing depth of 10.21× (Table S1).

The polymorphic sites within the dataset, as well as the number of r80 sites for the 12 test samples, were visualized using R software, as depicted in Figure S1. As the *M* value increased from five, the curve representing polymorphism sites gradually became stable, and the quantitative distribution of SNP sites on each locus tended to be balanced. Consequently, the operating parameters for the full sample dataset were set with *M* and *n* values of five. Subsequent sequence comparison and analysis with de novo-assembled contigs, along with filtering through the POPULATIONS module, removed biologically implausible and statistically insignificant sites. This rigorous process yielded a total of 1,930,419 SNP sites across 291 individuals, exhibiting a uniform distribution across the reads. Further refinement was conducted using VCFtools and PLINK software, resulting in a final dataset of 27,592 high-quality consensus SNP sites, which were then utilized for subsequent analyses.

### 2.2. Population Genetic Structure

**Figure 1:**
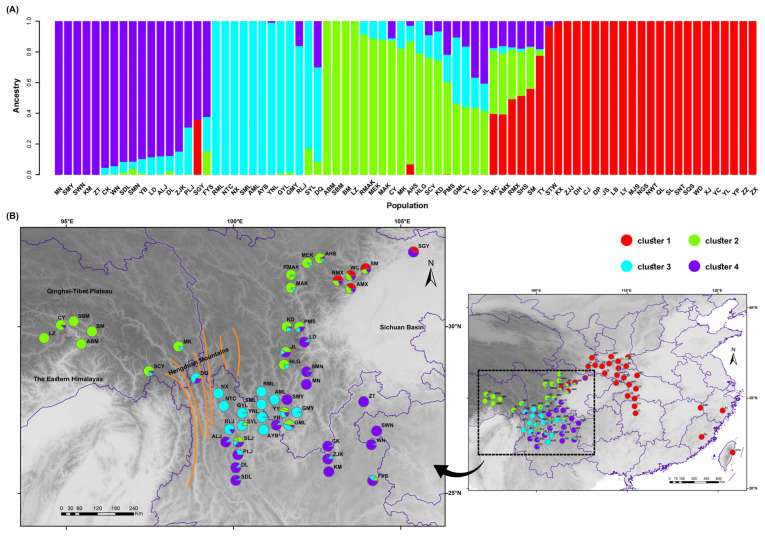
Optimal grouping results from ADMIXTURE. (**A**) Bar chart illustrating the best grouping results. (**B**) Map depicting the best clustering results.

Using the consensus dataset of 27,592 SNPs, ADMIXTURE software was used to analyze the genetic ancestry backgrounds of 76 populations. The optimal number of ancestral components was determined at *K* = 4, as indicated by the minimum CV error rate Figure S2. This finding suggests the existence of four distinct genetic clusters among the 76 populations. The optimal grouping determined by ADMIXTURE was used to visualize the ancestral proportions within each population using R software (Figure 1A). Each column represents a distinct population, with the varying lengths of the colored segments indicating the proportion of each ancestral component in its genome.

The optimal grouping results were integrated with the geographical locations of the populations and mapped accordingly (Figure 1B). The analysis indicates that these four genetic clusters exhibit a phenomenon of geographical aggregation. Overall, the populations were predominantly divided into two major regions: the eastern and western regions. The populations in the eastern region predominantly consisted of only one genetic component (cluster 1) and were primarily distributed in the Qinba Mountains and Central and East China regions, displaying a striped distribution pattern. The remaining three genetic clusters were predominantly concentrated in the western region of China and exhibited a hierarchical division from north to south. The populations in the Eastern Himalayas to the northwestern edge of the Sichuan Basin were primarily classified as cluster 2, while those in the Hengduan Mountains, as well as the populations in the southwestern edge of the Sichuan Basin and the Yunnan–Guizhou Plateau, were further subdivided into the other two subgroups (cluster 3 and 4).

Genetic structure testing of multiple species in sympatric distribution areas, such as eastern Tibet, the northwestern edge of the Sichuan Basin, and the Hengduan Mountains, revealed that sympatric species in these regions have the same or similar genetic components. For instance, the SBM, ABM, and BM populations, which belong to *Q. spinosa*, *Q. aquifolioides*, and *Q. guyavifolia*, respectively, were all collected from Bomi in Tibet. They possessed the same genetic components (cluster 2). Similarly, the AML population of *Q. aquifolioides*, the SML population of *Q. spinosa*, and the RML population of *Q. rehderiana* were all collected from the Mianya Mountains. They belong to a similar genetic component (cluster 3). Moreover, in regions where populations overlap several genetic clusters, there is a mixing of genetic components within the population. For example, the AMX and RMX populations, which belong to *Q. aquifolioides* and *Q. rehderiana*, respectively, are located in Mao County and at the intersection of three genetic clusters. These two populations share a similar genetic component and contain multiple ancestral genetic components (cluster 1, cluster 2, and cluster 4).

### 2.3. Phylogenetic Relationships

**Figure 2:**
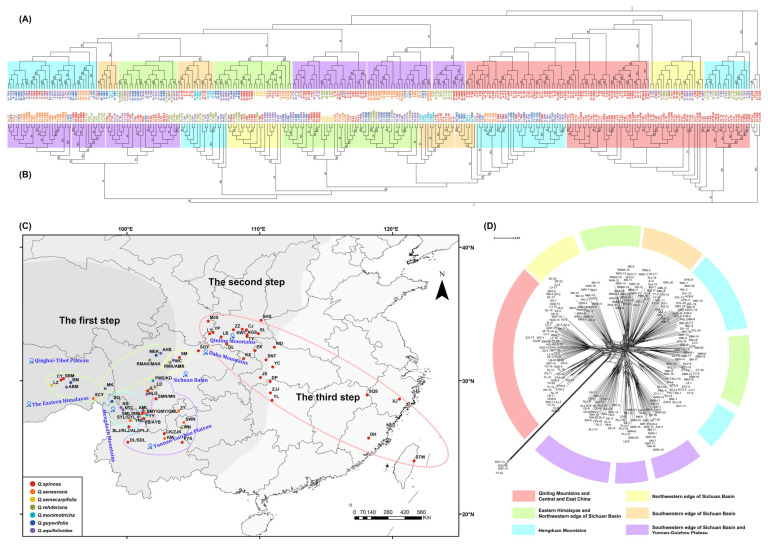
Phylogenetic tree reconstruction of 291 samples. (**A**) ML evolutionary tree. (**B**) NJ evolutionary tree. (**C**) Geographical distribution map of genetic clades. (**D**) SplitsTree splitting network diagram.

In this study, a fully resolved phylogeny of seven species was reconstructed using both the ML and NJ methods. The results revealed that the branching structures of the ML (Figure 2A) and NJ trees (Figure 2B) were congruent, and individuals were clustered into terminal branches of the evolutionary tree based on their affiliation with the respective populations.

Based on the geographic distribution of populations, the populations located in different geographic tiers of China constitute major branches on the evolutionary tree (Figure 2C). For example, the populations distributed in the Qinba Mountains and Central and East China were clustered into one branch, which is primarily located in the transition from the Second to the Third Geographical Step of China. The species within this branch were predominantly represented by *Q. spinosa*. The populations located in the Second Geographical Step of China, such as the Yunnan–Guizhou Plateau, and extending northward to the Sichuan Basin, were clustered into one branch based on genetic distance. The remaining branch populations were primarily located on the First Geographical Step of China and were further subdivided into three smaller branches based on geographical distance: the Eastern Himalayas branch, the Eastern Himalayas branch extending eastward to the edge of the Sichuan Basin, and the Hengduan Mountains branch. It is noteworthy that some populations in the Hengduan Mountains did not cluster genetically based on geographical proximity. The Chinese Geographic Steps exhibit a distinct geographical division in plant genetic clustering.

The evolutionary tree clustering results were generally concordant with the analysis of the ADMIXTURE genetic structure. From the perspectives of genetic clustering and the spatial distribution of evolutionary branches, the clustering of genetic branches in the eastern regions was relatively independent. In the western regions, the genetic clustering of populations reveals that those belonging to the same geographical step exhibit closer genetic relationships and can engage in gene flow, with evidence of introgression among the genetic components within populations. Populations from different geographical steps, even if they are geographically close in a straight line, exhibit distinct genetic components. Furthermore, the SplitsTree analysis (Figure 2D) indicated that the western clustering branches exhibited a process of reticulate evolution, with no distinct species boundaries in the genetic clustering of individuals. Populations and individuals from different species could be intermixed and clustered within a genetic lineage.

### 2.4. Gene Flow and Introgression

**Figure 3:**
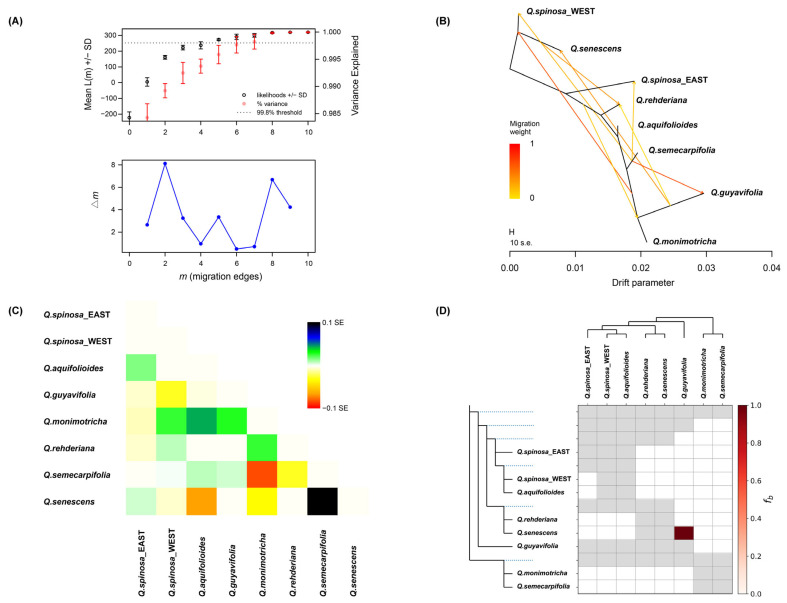
Gene flow results and gene introgression detection between species. (**A**) OptM results. (**B**) Gene flow events determined by TREEMIX software. (**C**) Results of the residual heatmap generated by the TREEMIX software. (**D**) Gene introgression between species.

Given the greater intraspecific differentiation within *Q. spinosa* than interspecific differentiation [27], the populations of *Q. spinosa* were grouped into eastern and western clusters based on genetic clustering to mitigate the influence of introgression bias. The OptM analysis (Figure 3A) indicated that the Δ*m* value was relatively high at *m* = 2 and *m* = 8. By correlating these results with the covariance matrix, *m* was set at eight as the optimal value for TREEMIX, a model that accounts for 99.8% of the data. Consequently, the analysis revealed eight historical gene flow events among the seven species (Figure 3B), indicating a complex pattern of reticulated gene flow. The covariance residual heatmap (Figure 3C) revealed predominantly positive residual standard deviations between the species, suggesting a closer genetic relationship between species than inferred from the ML tree and implying significant genetic introgression between them. Furthermore, the heatmap’s color scale approached zero, indicating that the model accurately reflected the actual covariance between species.

The f-branch diagram illustrates the extent of gene introgression between species (Figure 3D). The shade of the color blocks in the diagram represents the percentage of gene introgression, with darker shades indicating higher percentages. Figure 3D shows gene introgression among multiple species, particularly between *Q. guyavifolia* and *Q. senescens*. In conjunction with the TREEMIX results, a pronounced gene flow event between *Q. guyavifolia* and *Q. senescens* was identified.

### 2.5. Ecological Niche Differentiation and Ancestral Reconstruction

**Figure 4:**
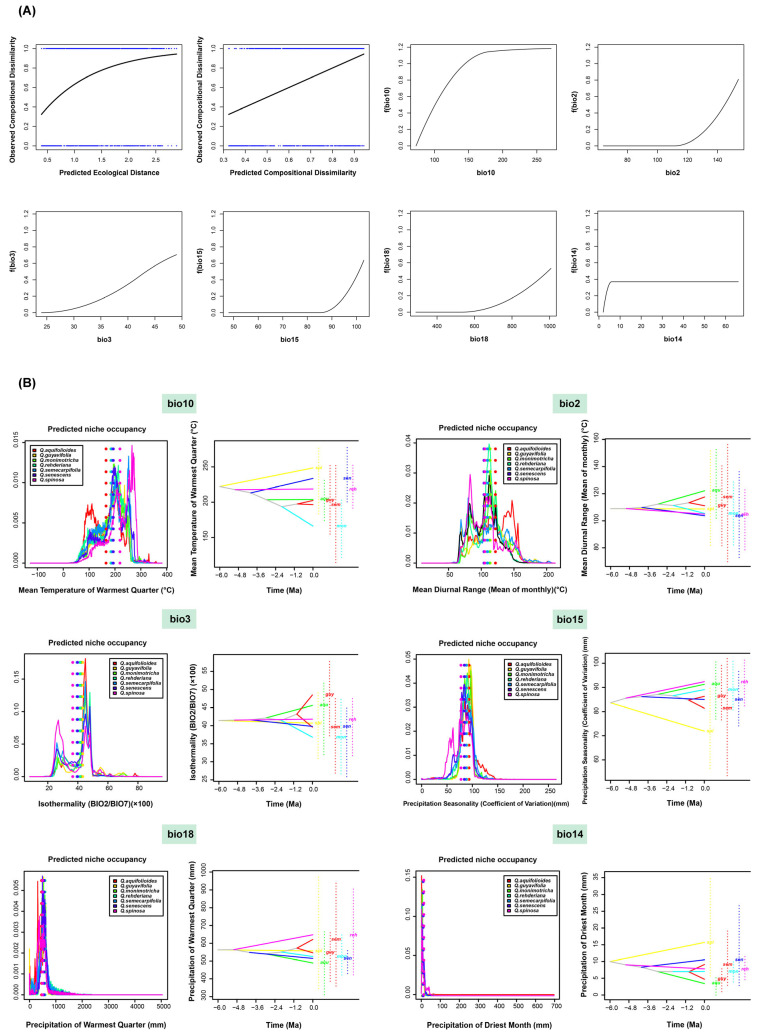
Driving forces of biodiversity. (**A**) Fitted generalized dissimilarity models. The shape of the spline indicates the magnitude of the total biological change along the gradient. (**B**) PNO profiles and inferred history of climatic tolerance evolution for seven species under climatic variation. The PNO horizontal axis represents the occupancy of various climatic indicators, while the vertical axis indicates the suitability of each species for each variable depicted.

Considering geographical distance and climatic parameters, the fitted splines for all significant environmental variables in the GDM were determined, and six major factors were plotted (Figure 4A). The factors with the greatest impact on the spatial biodiversity patterns of the seven species were bio10 (mean temperature of warmest quarter), bio2 (mean diurnal range), bio3 (isothermality), bio15 (precipitation seasonality), bio18 (precipitation of warmest quarter), and bio14 (precipitation of driest month). These results suggest that temperature plays a more significant role in the biodiversity distribution patterns of the seven species.

By integrating the SDM results for the seven species, Figure 4B illustrates the ecological niche occupancy and the reconstruction ancestral state of climate adaptation under the six most influential climatic factors affecting the biodiversity distribution patterns. The results indicated subtle heterogeneity in the occupation of each bioclimatic variable across the species. The dotted lines represent the weighted average niche occupancy for each species under each of the six climatic factors. As shown in Tables S2–S20, the upper-right and lower-left corners of the table represent the ecological niche similarity between species as calculated by Schoener’s D [28] and Hellinger distance values, respectively. Among them, the Schoener’s D value [29] for niche similarity under the six most influential climatic factors ranged from 0.59 to 0.91, with an average of 0.78. The results quantitatively reflect the differential impact of climatic factors on the niche occupancy of closely related species.

The reconstruction of the ancestral state of climate adaptation, depicted in Figure 4, revealed significant changes in climatic tolerance across the seven species, with each species exhibiting differential responses to climatic factors. According to the results from *phyloclim*, the seven species have experienced dispersive evolution under certain climatic conditions, for example, under the bio10 (mean temperature of warmest quarter). This indicates that the climatic tolerance of each evolutionary clade was distinct. Conversely, when the evolutionary clades showed a convergent clustering state under a certain climatic factor, this indicated that the climatic tolerance of the seven species tended to be uniform.

## 3. Discussion

### 3.1. Genetic Structure and Phylogenetic Relationships

Genome-wide SNP data were utilized to reconstruct the phylogenetic relationships of sclerophyll oaks using the NJ method and the ML method. The branch structures of the ML tree (Figure 2A) and the NJ tree (Figure 2B) were consistent. Both the NJ tree and the ML tree indicated that populations of different species were clustered into large clades based on geographic distance and similarity of genetic components, with no clear boundaries between species. The genetic clustering of the 76 populations was generally divided into two major branches, eastern and western, with significant genetic differentiation between the two. According to the spatial distribution of the two branches, the populations in the eastern branch were mainly from the Qinba Mountains and Central and East China regions, while the populations in the western branch were mainly from the Eastern Himalayas–Hengduan Mountains and their adjacent vast areas. The geographical gap between these two major lineages is around 105° E, which roughly coincides with the boundary between the Sino-Himalayan forest subkingdom and the Sino-Japanese forest subkingdom in the Sino-Japanese floristic region [30].

The uplift of the Tibetan Plateau has shifted China’s topography from an east-high and west-low configuration to a west-high and east-low landscape, creating a three-tiered terrain [31]. This shift has led to the longitudinal diversification of plant distributions [32]. Simultaneously, the Asian monsoon, influenced by geographical isolation, has created distinct environments, fostering the formation of phylogeographic breaks. For instance, the western subtropical region of China, west of approximately 105° E, is primarily influenced by the Indian Ocean monsoon, while the eastern region, east of approximately 105° E, is primarily affected by the East Asian monsoon. The contrasting climatic environments in the east and west have contributed to the formation of phylogeographic breaks around 105° E. The 105° E geographical boundary has been validated as a phylogeographic gap in various plant communities, including *Primula obconica* [33], *Euptelea*, and other deciduous broad-leaved forests [34,35], and *Quercus glauca*, *Castanopsis fargesii*, and other evergreen broad-leaved forests [36,37]. These studies consistently demonstrate the importance of the 105° E boundary as a significant phylogeographic gap within the Sino-Japanese floristic region.

The western branch of this study can be further subdivided into sub-branches, such as the Yunnan–Guizhou Plateau extending northward to the edge of the Sichuan Basin, the Eastern Himalayas branch, the Eastern Himalayas extending westward to the edge of the Sichuan Basin branch, and the Hengduan Mountains branch. The genetic structure results (Figure 1A) were generally consistent with the genetic clustering. There were no clear boundaries between species. The eastern branch contained only one genetic component (cluster 1), while the western region had three different genetic components. According to the geographical terrain, in the subtropical region of China, the eastern region is predominantly hilly. Even during the alternating glacial and interglacial periods of the Pleistocene, the eastern region had more opportunities for gene flow between populations, thus presenting a single genetic background. Conversely, the western region is characterized by numerous mountains and karst landscapes, leading to higher fragmentation and isolation, resulting in greater genetic differentiation and diversity [38].

Previous research has identified a continuous uplift of the Tibetan Plateau from the Miocene to the Pliocene [39,40], accompanied by a series of intense geological events and climatic fluctuations. Fossils of alpine oaks on Shishapangma Peak suggest that the Tibetan Plateau has risen by approximately 3000 m since the Pliocene. These changes have significantly influenced species differentiation patterns and fostered the evolution of local species [41]. Since the late Cenozoic, the Himalayas and Hengduan Mountains have undergone complex geological transformations, creating a variety of landforms and providing diverse habitats for plant communities. The Eastern Himalayas–Hengduan Mountains formed the alpine plant communities and are conducive to the growth of most sclerophyllous oaks. Meanwhile, these complex mountain systems have formed independent genetic units, with populations distributed like “islands” across mountain ranges, resulting in a diverse array of genetic compositions [42]. The exchange of genetic information between populations across different mountain ranges may be impeded by mountain barriers. Consequently, populations in the western branches have formed multiple different genetic clusters. Furthermore, the western branch of this study is primarily situated on the First and Second Geographical Steps of China. According to the geographical distribution, populations were distributed near the boundary between the First and Second Steps of the Chinese geographical terraces. Although the geographic distance between populations was close to a straight-line distance, their genetic clustering belongs to different branches. This phenomenon confirms that the Chinese Geographical Steps play a significant role as a geographical barrier in the genetic differentiation of species, hindering gene flow on both sides. Therefore, the genetic clustering of individuals is influenced by a multitude of factors, including genetic background and biogeographic boundaries.

The Hengduan Mountains serve as the cradle of alpine flora and as a vital habitat for alpine plant communities [43]. Field surveys have revealed that most sclerophyll oaks coexist sympatrically in eastern Tibet, the western Sichuan Basin, and the northern Hengduan Mountains [44]. This study demonstrates that sympatric species share similar genetic backgrounds. For instance, the populations of AML, SML, and RML collected from the Mianya Mountains in Sichuan are different species, but share a similar genetic component in sympatric regions. The evolution diagram for individuals developed by SplitsTree revealed the absence of distinct species boundaries (Figure 2D). The genetic relationships among all individuals exhibited a reticulate pattern. Populations and individuals of different species mingled and formed clusters in distinct geographical genetic clades. Each node in the SplitsTree network tree radiated outward during evolution, illustrating the intricate network of evolutionary processes within species. Furthermore, the TREEMIX detection indicated that pervasive and reticulated gene flow events occurred among the seven species (Figure 3B). To quantitatively assess interspecific introgression, Dsuite was used, and an f-branch diagram was generated for visualization (Figure 3D). The results demonstrated prevalent introgression among the seven sclerophyllous oak species.

Interspecies hybridization is a common phenomenon in nature, particularly among closely related species [45,46,47]. For instance, the genus *Picea* displays reticulate evolution in subtropical regions [48]. Radiation divergence has led to the formation of distinct species. Despite this, bidirectional gene flow persists due to overlapping distributions and synchronized flowering periods, facilitated by pollen dispersal. Natural hybridization is common within the *Quercus* genus [49]. For instance, changes in climatic conditions and landscape features, as well as human-induced disturbances like fire, have facilitated hybridization between two California white oaks with distinct ecological niches [50,51,52,53]. Additionally, the directionality of interspecific introgression between *Q. robur* and *Q. petraea* was assessed through genome-wide scans of SNP divergence. Despite pronounced genetic differentiation at outlier loci under selection, the research revealed ongoing bidirectional gene flow between these two oak species. Notably, historical asymmetric introgression from *Q. robur* to *Q. petraea* has been detected [54].

Hybridization and introgression have long played a key role in species formation and adaptive evolution in many plant groups [55,56]. For many wind-pollinated, widely sympatric forest tree species, adaptive introgression is also a major driver of their post-glacial expansion and adaptation to new environments [57]. An example of this is *Cupressus gigantea*, which originated from the Tibetan Plateau at higher latitudes and altitudes. It has utilized ancient introgression to help *Cupressus duclouxiana* expand into cooler and drier habitats following the retreat of the glaciers [58]. For *Quercus* plants, pollen dispersal is primarily wind-driven, with more pollen produced at lower altitudes, creating favorable conditions for genetic introgression among species [59].

Interspecific introgression evidence was detected in sympatric populations of *Q. acutissima* and *Q. variabilis* in East Asia. The study found that geographic distance and environmental similarity jointly determine the intensity of interspecific introgression. Moreover, populations with more similar environmental conditions had a higher probability of adaptive introgression occurring in the same location [60]. Sclerophyllous oaks have often been grouped into composite taxa with overlapping distributions, and natural hybridization between species within a composite group is widespread [61]. In this study, the flowering period of the seven species ranged from March to May. Their suitable growth areas largely overlap in the forested mountains of Southwest China. Considering the cross-pollination characteristics of *Quercus* plants [49], the gene exchange between species distributed in sympatric mountains is frequent, and interspecific hybridization can occur. When populations overlap in sympatric regions, there are opportunities for the exchange of genetic components between them. This sympatric overlap region provides an explanation for the occurrence of reticulate gene flow in sclerophyllous oaks.

### 3.2. Niche Differentiation and Adaptation

The range of species is limited by their capacity to adapt. When the environment imposes a strong filtering effect, closely related species tend to occupy similar habitats. For example, most species within the genus *Rhododendron* favor acidic soils on mountaintops, an example of phylogenetic aggregation. Variation in environmental resource use and niche differentiation is essential for species coexistence [62]. Research confirms that niche differentiation can be influenced by various resource dimensions, including soil texture, nutrient availability, salinity, and more [63,64]. Investigating the impact of different climatic factors on niche differentiation is crucial for understanding the coexistence and maintenance mechanisms within the seven species under study.

By integrating species potential distribution data with 19 climate factor variables, the R package ‘phyloclim’ was used to assess the niche occupancy of each species under varying climate dimensions. The degree of niche overlap was quantified using Schoener’s D value and the Hellinger distance. The results indicated that the spatial patterns of biodiversity for the seven sclerophyllous oaks are mainly influenced by six climatic factors. Among the six most influential climatic factors, the differences in niche similarity among the species were mainly reflected in temperature differences. Niche similarity under these six factors ranged from 0.59 to 0.91, with an average of 0.78. Despite their close phylogenetic relatedness, these seven species show both niche overlap and divergent ecological adaptations.

Sclerophyllous oaks, compared to broad-leaved tree species at lower altitudes, possess a unique ability to adapt to challenging ecological conditions at higher elevations and seek advantageous habitat conditions. However, with increasing altitude, the temperature decreases. Consequently, there are differences in the altitude ranges that are suitable for the species within the group. According to the actual field research (Table S21), *Q. aquifolioides* frequently thrives on adret slopes and within alpine pine forests at altitudes of 2000–4500 m, where it is a constructive and dominant species in high-altitude regions. *Q. rehderiana*, on the other hand, has a more limited suitable altitude range and is generally sparsely distributed within mid-altitude areas of 1500–4000 m. *Q. semecarpifolia* predominantly occurs on the slopes or valleys of oak-pine forests at altitudes of 2600–4000 m. *Q. guyavifolia* is well-suited to grow on oak and pine forest slopes at altitudes of 2500–3900 m. *Q. senescens* thrives on hillside slopes at an altitude of 1900–3300 m. *Q. monimotricha* thrives on sunny slopes or ridges at an altitude of 2000–3500 m and is the dominant species in the krummholz region. Despite overlapping altitudinal ranges among the species, empirical studies have revealed that sclerophyllous oaks exhibit significantly varying temperature and light requirements, such as shady or full sun requirements in hillside habitats.

Previous studies have indicated that environmental factors, such as altitude and slope, play a pivotal role in determining the ecological niches of species within the Qinling pine–oak forest [65]. As the altitude decreases and the slope increases, the 29 dominant species in the plant community exhibit diverse distribution patterns along this gradient, indicating that competition for available resources among species in mixed forests is not intense. Furthermore, by quantifying ecological data from quadrat units categorized by altitude, slope, and aspect, the study concluded that niche changes and neutral evolution significantly contribute to the formation of subtropical evergreen broad-leaved forest communities [66]. Consequently, despite the breakdown of species boundaries and the lack of complete reproductive isolation between sympatric species, niche differentiation remains a critical factor in maintaining species boundaries, as observed in the seven species of this study. Moreover, the wide elevational range of *Q. spinosa* (900–3000 m) correlates with its broad niche breadth, while the pronounced niche differentiation suggests adaptive divergence from sympatric species. Niche width represents the scale of resource utilization by plants [67]. A larger value signifies a species’ greater ability to utilize resources and a broader population distribution. Conversely, the degree of species specificity is high, with each species adapting to specific regions. This may also be a crucial reason for the widespread distribution of *Q. spinosa*.

The habitat adaptability of species is a pivotal factor in preserving local differentiation. An investigation into altitude in the Qinling Mountains posited that habitat heterogeneity was the predominant factor contributing to the long-term coexistence of species within specific spatial regions [68]. The predominant mechanism for the coexistence of species is the disparity in their habitats [69]. A critical aspect of local adaptation is its potential to initiate, facilitate, and catalyze speciation. Ecological speciation hypotheses specifically suggest that speciation may be a byproduct of local adaptation. It was shown in this study that the climate tolerances of the species exhibited distinct responses during the reconstruction of ancestral state climate adaptability. The dispersion of climate tolerance within subclades of the evolutionary tree indicates that the climatic tolerance of each subclade tends to vary, particularly under the influence of the six most significant climatic factors that affect the spatial patterns of biodiversity. Therefore, we contend that although these seven species are closely related within the group, they exhibit a certain degree of niche overlap while still retaining some differences. The differences in climatic tolerance among species may also contribute to maintaining species boundaries.

## 4. Materials and Methods

### 4.1. Sample Materials

This study selected seven sclerophyllous oak species primarily distributed in China. The sampled materials of these seven species cover the majority of the sampling sites recorded in the CVH China Digital Herbarium, with specific morphological differences detailed in Table S22. The formal identification of the samples was performed by Gui-fang Zhao (Northwest University).

During sample collection, the distance between individuals within each population was maintained at least 100 m. Detailed records of plant material collection sites and sources are provided in Table S21. In total, this study collected 291 samples from 76 populations, including 130 individuals from 38 populations of *Quercus spinosa*, 50 individuals from 13 populations of *Quercus aquifolioides*, 33 individuals from 7 populations of *Quercus rehderiana*, 33 individuals from 7 populations of *Quercus guyavifolia*, 9 individuals from 3 populations of *Quercus monimotricha*, 5 individuals from 1 population of *Quercus semecarpifolia*, and 31 individuals from 7 populations of *Quercus senescens*. Additionally, *Fagus engleriana* was included as an outgroup. Although sample sizes were limited in some locations, the large number of polymorphic SNPs derived from the whole-genome sequencing ensured robust estimates of genetic diversity. Consequently, the small sample size is unlikely to bias within-population genetic diversity, as previously reported [70].

Fresh leaves were collected in the field and preserved in silica gel for drying. All sampled materials did not include any endangered or protected species, and collections were conducted in compliance with the relevant institutions, national and international guidelines, and legislation. Voucher specimens have been deposited in the Key Laboratory of Resource Biology and Biotechnology in Western China (KL-RBBWC), Ministry of Education, at Northwest University.

### 4.2. DNA Extraction and Detection

The entire genomic DNA from the leaf samples was isolated using a modified CTAB method and a commercial DNA extraction kit [71]. The quality and concentration of the extracted genomic DNA were assessed through 1% agarose gel electrophoresis and quantified using a NanoDrop 2000 UV spectrophotometer (NanoDrop, Wilmington, DE, USA). The genomic DNA was then diluted to an 18 ng/μL concentration and stored in TE solution.

### 4.3. Library Preparation for SLAF Sequencing

The SLAF library was prepared in accordance with the protocols outlined by Sun, with minor adaptations [26]. Genomic DNA from the samples was digested with the *RsaI* restriction enzyme, and the accuracy of the enzyme digestion was validated using *Oryza sativa*. Following PCR amplification, purification, and gel electrophoresis on a 2% agarose gel, DNA fragments of approximately 314–414 base pairs (including indexes and adaptors) were isolated and diluted. These fragments were then sequenced on the Illumina HiSeqTM 2500 platform (Illumina, Inc.; San Diego, CA, USA) at Beijing Biomarker Technologies Corporation. A dual-indexing strategy [72] was employed to identify the raw sequencing data and obtain the reads for each sample. After removing the adapter sequences, the sequencing quality was assessed based on the guanine–cytosine (GC) content and the Q30 value (Q_-score_ = −10 × log_10_*P*; indicating a 0.1% chance of an error and thus 99.9% confidence). To maintain the quality of the sequencing, all paired-end reads were trimmed to a length of 125 bp×2.

### 4.4. Development of Consistent SNP Loci

Genotyping of all individuals was conducted using the STACKS-1.47 software pipeline [73]. Twelve individuals were randomly selected as test samples for parameter optimization. The test procedure ensured that SLAF-tags with a minimum depth of stacks were retained, with *m* set to three. The USTACKS and CSTACKS programs were executed using the optimal parameters, ensuring the identification of polymorphism loci present in at least 80% of individuals from each population. After parameter tuning, the USTACKS program was used for de novo assembly of paired-end sequencing data without reference genomes, with *m* set to three and *M* set to five. The CSTACKS program (with *n* set to five) was utilized to construct a catalog, which was then matched against each sample to identify alleles using SSTACKS. The POPULATIONS module consolidated the catalog of datasets for all individuals, resulting in the generation of consensus sequences. The SNP dataset underwent formatting and filtering using VCFtools (v0.1.16) software [74] and PLINK1.7 [75]. Low-quality and non-statistically significant sites were eliminated based on the following criteria: integrity (GENO) > 0.6, minor allele frequency (MAF) < 0.05, and Hardy–Weinberg equilibrium (HWE) *p* < 10^−4^. Loci that included all populations and were successfully genotyped in at least 90% of individuals were retained for subsequent analyses.

### 4.5. Analysis of Genetic Structure

Population genetic structure aids in the comprehension of speciation and the identification of genetic clusters through genotype and phenotype association studies. ADMIXTURE (v1.3.0) software [76] employed the maximum likelihood (ML) clustering algorithm to infer individual ancestry from a multi-locus SNP genotype dataset, estimating the number of genetic clusters (*K*) from a predefined number of ancestral populations without the need for a priori population designation. A series of tests were conducted with *K* values ranging from 1 to 10, and the optimal number of individual groups was determined based on the cross-validation (CV) error rate.

### 4.6. Molecular Phylogenetic Tree

To gain a comprehensive understanding of the phylogenetic relationships and evolutionary pathways among species and individuals, three methods were employed to reconstruct the phylogenetic relationships and construct a network dendrogram for the seven species. Utilizing the consensus SNP dataset, the evolutionary tree was constructed using MEGA 11 software [77], with the neighbor-joining (NJ) and ML methods employed for comparative analysis of the same or similar evolutionary branches. The best nucleotide substitution model was selected in advance for constructing the ML tree. Following the results of the Akaike information criterion (AIC) (Table S23), the nucleotide substitution model was set to GTR + G + I, and the bootstrap method was utilized to assess branch support rates (replicates = 1000). The NJ tree was constructed using the Kimura two-parameter replacement model, with the bootstrap method employed to test branch support rates (replicates = 1000).

SplitsTree4 software [78] was employed to generate a split network diagram, which utilized the p-distance model and the neighbor-net method to construct an evolutionary network. The resulting evolutionary tree was subsequently edited using the online interaction tree of life (ITOL) website (http://itol.embl.de/, accessed on 30 July 2025).

Species trees were reconstructed using the SVDquartets [79] implemented in PAUP* v.4.0 [80]. This approach is capable of handling simplified genome data with a substantial number of missing sites [81].

### 4.7. Gene Flow and Introgression Detection

Building upon allele frequency data from multiple populations, TREEMIX (v1.13) software [82] was used to construct an ML tree and infer population splitting and mixing patterns. The historical mixing and gene flow events were calibrated on the phylogenetic tree. For the TREEMIX analysis, the range of *m* values was set from 1 to 10. To account for multiple cycles, the OptM algorithm was employed to select the optimal *m* value, representing the number of potential gene flow events between species given a certain amount of gene flow, and the fitting degree of the model was assessed through a residual heatmap constructed from the interspecies covariance matrix.

D-statistics, also known as ABBA–BABA statistics, are commonly used to evaluate evidence of gene flow between populations or related species [83]. Using the fast C++ program, Dsuite (v0.5) software [83] was used to calculate the D-statistic for numerous population and species combinations, and the extent of introgression at specific sites was determined by evaluating the proportions of mixtures. The resulting files were visualized in the f-branch diagram through the dtools.py script.

### 4.8. Ecological Niche Differentiation

The ecological niche represents the spatial and temporal position of a population within an ecosystem and its functional relationship with other populations. MaxEnT 3.3 software [84] was used to simulate and predict the potential distribution areas of each species. The natural geographical distribution of each species was obtained from the Global Biodiversity Information Facility [85]. To mitigate the impact of spatial autocorrelation on the analysis, duplicate sample points and sites with a longitude–latitude distance of less than 0.4° were excluded. The final specimens included 175 records of *Q. spinosa*, 91 records of *Q. aquifolioides*, 114 records of *Q. rehderiana*, 74 records of *Q. guyavifolia*, 59 records of *Q. monimotricha*, 74 records of *Q. semecarpifolia*, and 63 records of *Q. senescens*.

Species ecological niche overlap was calculated based on species distribution models (SDM) generated with MaxEnT, combined with the Probability of Niche Overlap (PNO) function in the R package ‘phyloclim’ (v0.9.5) [86,87]. The weighted average of niche similarity between species was derived from 1000 bootstrap samples of the PNO profiles. By combining ecological niche models with a species tree, the R package ‘phyloclim’ was used to reconstruct ancestral niches and quantify niche evolutionary rates across clades. The ecological states of ancestral nodes were inferred using maximum likelihood estimation, based on the niche characteristics of extant species represented as terminal taxa in the phylogeny.

Generalized dissimilarity modeling (GDM) is a powerful and unique statistical method designed to describe and predict changes in the spatial patterns of biodiversity across space, time, and environmental gradients [88]. In this study, the geographical information of 76 populations along with 19 bioclimatic variables was modeled. Climate data were obtained from WorldClim (https://www.worldclim.org, accessed on 30 July 2025). The R package ‘gdm’ (v1.5.0-9.1) [88] was implemented to quantify the relative contributions of environmental gradients and geographic distance to beta diversity patterns across the study region. By fitting non-linear response curves to species turnover along environmental and spatial gradients, this approach disentangles the complex drivers of biodiversity variation.

## 5. Conclusions

Our study elucidates the intricate evolutionary processes governing the coexistence and diversification of seven Chinese sclerophyllous oaks, leveraging integrated genomic and ecological analyses. A principal finding is the identification of a major genetic divergence line approximating 105° E longitude, which demarcates eastern and western lineages. This biogeographic boundary correlates strongly with floristic zones delineated by contrasting monsoon-driven climatic regimes. Our results indicate that the high topographic complexity in western regions facilitates elevated levels of genetic diversity, potentially through population fragmentation and localized adaptation. Conversely, eastern populations exhibit greater genetic homogeneity, suggesting extensive, stable gene flow across more contiguous habitats. Furthermore, our analyses reveal pervasive interspecific hybridization and reticulate evolution, particularly among geographically overlapping populations, contributing to genetic admixture and potentially challenging traditional species delimitation. Notably, each species occupies distinct ecological niches, with temperature-related environmental gradients proving particularly critical for mediating coexistence, likely through stabilizing selection. These observations underscore a dynamic interplay between neutral evolutionary processes, notably gene flow, and selective pressures, specifically niche differentiation, acting synergistically to drive lineage diversification within this rapidly radiating genus. Our work thus provides specific, in-depth insights into the evolutionary dynamics of these sclerophyllous oaks, emphasizing the utility of multi-omics and ecological niche modeling approaches for unraveling species coexistence patterns in topographically heterogeneous regions.

## Data Availability

The datasets used and analyzed for this study can be found in the NCBI database with accession number PRJNA1104405.

## Supplementary Materials

The following supporting information can be downloaded at: https://www.mdpi.com/article/10.3390/plants14152403/s1, Table S1: Sequencing quality results summary for individual samples. Table S2–S20: Niche similarity detection of seven species under a single climatic variable (bio_1–bio_19). Table S21: Detailed information on the 76 population sampling sites. Table S22. Detailed morphological differences among the seven species. Table S23: Maximum likelihood fits of 24 different nucleotide substitution models. Figure S1: Parameter tuning of the test data. (A) The number of r80 sites corresponding to different *M* values. (B) Quantitative distribution of SNP sites on different loci. Figure S2: Cross-validation error rate results for 76 populations. Figure S3: PNO profiles and inferred history of climatic tolerance evolution for seven species under climatic variation. The PNO horizontal axis represents the occupancy of various temperature indicators, while the vertical axis indicates the suitability of each species for each variable depicted. Figure S4: PNO profiles and inferred history of climatic tolerance evolution for seven species under climatic variation. The PNO horizontal axis represents the occupancy of various precipitation indicators, while the vertical axis indicates the suitability of each species for each variable depicted.
