# Supplementary material for: Integrative Population Genomics Reveals Niche Differentiation and Gene Flow in Chinese Sclerophyllous Oaks (Quercus Sect. Ilex)
Source: Plants (Basel). 2025 Aug 3;14(15):2403. doi: 10.3390/plants14152403 (PMC12349489; doi:10.3390/plants14152403)
Supplement: Supplementary file 1 [file plants-14-02403-s001.zip › plants-3729462-supplementary.pdf]

Table S1. Sequencing Quality Results Summary for Individual Samples

Sample ID	Total Reads	Q30 Percentage (%)	GC Percentage (%)	SLAF number	Total depth	Average depth
ABM-14	3,111,411	89.95	41.92	145,647	2,063,177	14.17
ABM-16	3,376,298	91.51	39.94	150,486	2,148,142	14.27
ABM-18	2,087,374	93.04	40.63	114,816	1,298,941	11.31
ABM-19	1,718,265	93.04	40.38	123,971	1,149,751	9.27
ABM-9	3,604,004	93.4	38.86	144,619	2,347,671	16.23
AHS-10	1,454,974	88.69	40.54	118,783	864,041	7.27
AHS-13	1,423,202	89.37	39.63	99,094	896,237	9.04
AHS-15	1,856,457	88.46	39.33	107,805	1,119,751	10.39
ALJ-11	2,003,186	89.54	42.21	105,845	1,297,077	12.25
ALJ-13	2,798,968	93.58	40.07	145,486	1,881,005	12.93
ALJ-15	2,669,379	89.97	43.23	126,966	1,758,628	13.85
ALJ-19	2,710,416	90.32	42.51	133,344	1,811,041	13.58
ALJ-9	4,711,416	92.63	39.35	188,932	3,097,327	16.39
AML-10	1,746,337	93.21	38.48	112,919	1,035,994	9.17
AML-2	2,454,257	93.27	41.06	150,108	1,679,369	11.19
AML-3	2,598,103	92.96	40.38	148,430	1,755,836	11.83
AML-6	2,516,606	93.37	40.36	133,148	1,681,219	12.63
AML-9	2,894,834	93.16	38.47	140,671	1,958,662	13.92
AMX-10	3,108,358	93.47	41.22	175,093	2,036,205	11.63
AMX-11	1,604,138	88.11	40.22	108,640	967,154	8.9
AMX-16	2,884,786	93.19	41.03	144,346	1,855,083	12.85
AMX-17	4,889,388	89.03	39.8	156,386	3,121,007	19.96
AMX-19	2,211,423	89.06	40.15	138,141	1,361,476	9.86
AYB-11	2,788,311	93.13	40.12	157,545	1,768,047	11.22
AYB-2	3,858,535	89.77	40.64	143,985	2,623,518	18.22
AYB-31	2,777,808	88.7	39.66	138,535	1,715,745	12.38

AYB-4	3,797,619	89.79	39.75	144,153	2,539,376	17.62
AYB-9	2,919,699	93.35	40.59	152,173	1,913,111	12.57
BM-14	2,447,868	93.35	40.84	148,421	1,618,509	10.9
BM-17	2,852,080	93.06	40.96	142,676	1,663,722	11.66
BM-19	2,656,733	93.13	39.36	149,745	1,762,717	11.77
BM-20	2,931,940	92.98	41.36	136,658	1,891,250	13.84
BM-9	2,612,802	89.89	41.02	136,000	1,734,621	12.75
CJ-10	3,086,668	93.22	41.11	158,016	1,989,472	12.59
CJ-4	2,334,884	93.13	41.01	146,107	1,559,034	10.67
CJ-7	2,721,075	92.8	40.57	132,795	1,660,590	12.5
CK-11	3,378,500	89.83	39.69	132,224	2,250,356	17.02
CK-16	2,987,578	93.24	40.1	164,512	1,868,624	11.36
CK-20	3,660,111	93.1	41	157,654	2,466,505	15.65
CK-5	3,392,458	93.35	41.49	156,130	2,254,169	14.44
CK-6	3,124,035	93.07	42.61	142,537	1,894,531	13.29
CY-14	2,993,864	93.67	41.29	175,008	1,839,240	10.51
CY-19	2,278,921	93	41.95	125,729	1,421,197	11.3
CY-7	3,421,710	93.07	42.9	160,830	2,197,920	13.67
CY-8	2,974,467	90.18	40.59	132,289	2,002,379	15.14
CY-9	2,361,325	92.97	40.98	139,723	1,467,959	10.51
DH-14	2,345,063	88.35	40.19	133,223	1,395,104	10.47
DH-16	1,125,840	88.51	39.61	115,849	626,618	5.41
DH-8	1,798,961	89.72	41.43	108,796	1,181,444	10.86
DL-2	2,389,707	93.34	41.84	129,207	1,434,875	11.11
DL-5	2,624,740	92.81	41.05	140,992	1,666,376	11.82
DL-8	3,356,774	93.23	40.11	160,242	2,020,510	12.61
DP-2	1,673,015	88.41	40.07	129,084	1,012,302	7.84
DP-23	2,068,760	93.25	41.1	147,360	1,212,967	8.23
DP-5	1,480,873	88.85	39.85	104,457	933,981	8.94

DQ-1	2,073,439	92.92	41.31	128,176	1,352,782	10.55
DQ-12	2,135,495	93.41	40.63	146,250	1,362,577	9.32
DQ-9	2,837,715	93.63	41.27	153,708	1,908,641	12.42
FYS-11	2,170,421	90.19	40.38	121,079	1,448,606	11.96
FYS-15	1,836,037	92.99	41.38	132,488	1,128,438	8.52
FYS-20	2,682,653	93.1	40.86	160,620	1,641,197	10.22
GML-1	1,567,148	90.23	39.41	104,250	1,052,280	10.09
GML-11	1,727,408	89.56	39.69	111,191	1,124,138	10.11
GML-20	1,456,196	88.34	40.78	98,284	886,527	9.02
GML-3	2,151,492	92.88	41.09	131,292	1,393,424	10.61
GML-5	2,467,095	89.61	38.98	128,420	1,540,426	12
GMY-11	1,885,643	90.38	39.53	111,867	1,274,090	11.39
GMY-13	1,647,293	92.94	41.16	111,293	954,363	8.58
GMY-14	2,119,889	93.45	39.93	136,803	1,278,497	9.35
GMY-17	1,436,817	90.02	40.32	102,554	961,027	9.37
GMY-20	2,008,300	93.41	39.26	147,509	1,233,857	8.36
GYL-12	2,579,821	93.28	40.53	158,693	1,678,369	10.58
GYL-16	2,384,632	92.4	40.4	151,646	1,441,378	9.5
GYL-18	2,657,974	92.9	39.34	131,342	1,595,920	12.15
GYL-20	2,323,954	88.5	39.55	116,354	1,431,929	12.31
GYL-6	2,130,558	93.5	40.65	143,702	1,439,891	10.02
HLG-10	2,377,455	93.38	38.88	131,829	1,602,796	12.16
HLG-11	1,954,511	87.85	39.17	111,679	1,146,452	10.27
HLG-3	1,942,756	92.96	39.53	123,606	1,266,959	10.25
JL-2	1,598,796	89.41	39.95	105,927	1,009,817	9.53
JL-4	2,190,596	92.41	40.14	124,487	1,379,802	11.08
JL-9	2,309,913	92.43	40.45	121,370	1,322,792	10.9
JS-15	2,957,180	93.61	40.45	147,458	1,775,978	12.04
JS-18	2,449,911	89.07	39.96	131,638	1,571,182	11.94

JS-4	1,854,213	93.62	41.08	162,565	1,251,931	7.7
KD-12	2,262,059	93.41	41.32	156,697	1,480,805	9.45
KD-13	1,991,484	89.04	40.68	118,281	1,268,072	10.72
KD-3	1,708,288	94.06	41.81	151,594	1,099,951	7.26
KM-13	1,793,532	93.25	42.26	152,336	1,125,428	7.39
KM-3	2,164,764	93.34	41.89	166,508	1,418,862	8.52
KM-4	2,180,279	93.92	42.37	181,810	1,399,459	7.7
KX-1	2,293,852	93.57	41.45	163,544	1,542,956	9.43
KX-2	2,243,753	93.25	41.39	157,641	1,464,972	9.29
KX-3	1,882,483	93.54	41.21	164,434	1,097,312	6.67
LB-11	2,132,272	89.87	39.82	124,952	1,415,464	11.33
LB-13	2,213,088	93.75	40.22	158,998	1,514,943	9.53
LB-14	2,358,356	93.45	41.09	157,203	1,615,718	10.28
LD-3	1,016,615	88.28	40.47	84,421	595,106	7.05
LD-5	1,353,005	88.98	40.64	96,787	820,902	8.48
LD-6	1,209,581	87.87	42.7	98,851	653,876	6.61
LY-16	2,207,350	93.45	41.5	160,495	1,496,240	9.32
LY-17	1,878,433	93.48	42.17	159,768	1,217,382	7.62
LY-5	1,886,654	93.62	40.69	148,430	1,253,112	8.44
LZ-1	2,263,249	90.04	40.88	118,811	1,519,613	12.79
LZ-3	1,781,063	93.56	41.52	143,576	1,133,509	7.89
LZ-4	1,661,243	93.12	40.78	141,570	1,087,279	7.68
LZ-6	3,640,080	90.2	40.66	140,795	2,477,602	17.6
LZ-8	3,238,534	91.08	38.73	160,537	2,011,507	12.53
MAK-10	1,868,726	88.71	40.56	115,961	1,152,903	9.94
MAK-11	1,733,056	88.31	40.47	112,671	1,049,992	9.32
MAK-12	2,326,609	91.61	40.53	154,557	1,384,219	8.96
MAK-3	2,084,448	89.48	39.88	122,617	1,316,157	10.73
MEK-12	2,675,647	88.81	41.02	131,815	1,655,256	12.56

MEK-13	1,246,959	88.52	40.57	92,080	759,298	8.25
MEK-14	1,398,083	88.62	40.92	98,492	855,070	8.68
MEK-15	1,215,307	89.31	40.92	94,705	759,247	8.02
MEK-16	1,561,561	89.58	39.35	108,470	983,252	9.06
MJS-10	4,884,615	89.77	39.22	166,372	3,239,435	19.47
MJS-13	1,923,512	93.06	41.57	151,185	1,277,556	8.45
MJS-14	1,335,439	89.64	40.37	104,504	866,247	8.29
MK-11	1,733,494	89.35	39.79	111,009	1,078,112	9.71
MK-20	1,897,781	89.65	40.82	130,273	1,231,745	9.46
MK-6	2,321,970	93.86	39.76	158,556	1,528,308	9.64
MN-1	2,759,778	93.51	41.61	187,530	1,761,367	9.39
MN-11	2,369,474	94.18	40.27	151,589	1,645,433	10.85
MN-14	1,790,814	93.55	41.99	151,276	1,186,509	7.84
MN-5	2,189,986	91.26	40.47	133,074	1,340,987	10.08
MN-7	2,066,885	93.77	42.44	175,796	1,368,562	7.78
NGS-13	2,440,716	93.48	41.35	177,863	1,660,199	9.33
NGS-6	2,379,433	92.6	40.19	154,471	1,574,144	10.19
NGS-7	2,522,516	93.77	39.71	156,105	1,743,610	11.17
NTC-2	2,339,196	91.11	40.69	132,529	1,429,320	10.79
NTC-6	3,175,153	91.27	40.11	158,157	1,956,476	12.37
NTC-9	2,206,621	93.97	41.98	173,271	1,491,108	8.61
NWT-13	1,757,770	92.94	39.92	153,417	1,119,552	7.3
NWT-16	1,806,500	90.23	42.18	153,654	1,180,703	7.68
NWT-18	2,065,720	90.58	41.32	152,002	1,378,412	9.07
NX-1	2,206,381	93.39	38.39	161,482	1,379,409	8.54
NX-3	2,517,947	93.29	41.88	178,369	1,651,813	9.26
NX-4	2,861,662	92.83	41.68	190,358	1,880,698	9.88
PLJ-12	1,854,869	89.54	42.62	131,323	1,153,592	8.78
PLJ-15	1,158,026	87.86	40.89	89,605	675,792	7.54

PLJ-16	1,867,501	90.49	40.42	130,798	1,224,105	9.36
PLJ-17	1,510,145	87.8	41	106,591	853,940	8.01
PLJ-18	3,101,826	93.48	41.15	201,287	2,079,658	10.33
PMS-11	2,251,006	88.18	41.49	133,086	1,306,786	9.82
PMS-18	2,144,787	89.08	40.9	126,632	1,309,167	10.34
PMS-5	2,433,922	89.25	41.32	153,331	1,518,850	9.91
QL-13	1,509,954	89.73	41.39	129,928	998,769	7.69
QL-19	1,887,701	93.99	41.92	173,235	1,271,346	7.34
QL-20	1,892,445	90	40.63	152,858	1,243,835	8.14
RLJ-14	1,939,543	89.36	41.67	139,280	1,252,271	8.99
RLJ-15	1,880,568	94.29	42.32	173,945	1,266,069	7.28
RLJ-18	2,670,617	93.24	39.87	151,448	1,763,136	11.64
RLJ-2	1,838,765	93.79	41.81	161,538	1,193,830	7.39
RLJ-9	2,148,926	89.59	41.74	134,997	1,414,018	10.47
RMAK-12	1,962,333	88.45	40.64	115,520	1,152,271	9.97
RMAK-15	3,751,024	93.53	39.39	167,237	2,564,791	15.34
RMAK-19	1,034,009	89.24	42.8	92,780	643,115	6.93
RMAK-4	1,283,400	89.74	42.58	97,620	830,773	8.51
RMAK-5	1,552,823	89.7	40.79	110,724	1,006,989	9.09
RML-1	2,760,149	91.84	41.2	178,217	1,694,460	9.51
RML-12	1,752,704	94.21	40.18	162,503	1,201,588	7.39
RML-19	1,938,135	94.04	40.09	162,293	1,333,733	8.22
RML-6	1,655,415	94.2	41.37	152,513	1,121,160	7.35
RML-9	1,780,178	94.12	41.99	169,741	1,192,410	7.02
RMX-11	1,578,403	94.36	42.09	166,023	1,047,211	6.31
RMX-13	1,157,155	89.55	42.29	105,634	740,136	7.01
RMX-14	1,219,582	88.78	40.91	103,911	775,991	7.47
RMX-2	1,799,749	93.81	41.93	165,686	1,085,868	6.55
RMX-6	1,887,167	89.27	40.78	134,496	1,212,238	9.01

SBM-14	1,682,579	93.35	41.42	149,430	1,115,275	7.46
SBM-16	1,940,579	91.92	38.15	140,002	1,185,230	8.47
SBM-17	2,046,378	93.33	40.91	166,716	1,345,375	8.07
SBM-19	1,888,648	92.82	41.74	164,875	1,209,139	7.33
SBM-3	1,600,784	92.64	41.85	148,961	1,024,342	6.88
SCY-15	1,628,993	93.78	42.45	149,924	1,068,774	7.13
SCY-17	2,425,963	91.83	40.21	168,870	1,472,052	8.72
SCY-5	1,932,142	88.86	43.25	123,055	1,236,088	10.05
SCY-6	2,960,625	88.9	41.73	147,702	1,913,543	12.96
SCY-9	1,782,297	90.91	42.98	138,924	1,129,754	8.13
SDL-12	2,212,863	93.06	41.99	160,993	1,411,729	8.77
SDL-15	1,835,131	89.38	41.16	128,913	1,182,398	9.17
SDL-9	2,147,737	92.42	40.96	168,951	1,318,503	7.8
SGY-13	2,248,577	93.86	40.3	138,326	1,415,905	10.24
SGY-15	2,330,250	87.83	38.89	110,903	1,321,248	11.91
SGY-18	2,089,685	94.05	40.77	147,002	1,269,201	8.63
SHS-1	2,184,108	92.35	41.28	170,113	1,337,907	7.86
SHS-2	1,888,460	89.58	41.59	133,407	1,216,184	9.12
SHS-3	2,712,376	89.97	40.91	152,241	1,769,869	11.63
SL-10	1,935,330	93.51	41.03	161,295	1,162,671	7.21
SL-17	2,332,286	92.93	39.95	152,552	1,547,500	10.14
SL-9	1,767,185	93.46	41.97	159,514	1,125,709	7.06
SLJ-1	2,413,623	88.72	40.34	134,211	1,517,671	11.31
SLJ-11	2,169,656	88.72	40.64	128,119	1,334,506	10.42
SLJ-15	2,966,399	89.54	40.91	150,854	1,879,339	12.46
SLJ-17	2,738,167	88.91	40.48	152,809	1,733,583	11.34
SLJ-18	1,964,352	89.35	41.39	133,286	1,266,507	9.5
SM-10	1,868,924	93.34	40.85	135,465	1,247,412	9.21
SM-13	2,706,909	93.06	40.8	158,586	1,784,585	11.25

SM-14	2,807,301	93.06	40.47	155,269	1,886,406	12.15
SM-3	2,448,625	93.83	40.85	165,000	1,636,365	9.92
SM-7	3,810,484	93.03	40.33	182,469	2,585,243	14.17
SML-1	2,762,815	93.4	39.8	161,830	1,847,872	11.42
SML-2	3,166,082	93.71	41.01	171,593	2,156,922	12.57
SML-3	2,058,048	93.43	40.5	145,273	1,415,403	9.74
SML-6	1,962,754	92.94	41.24	134,691	1,324,608	9.83
SML-8	6,844,685	93.19	38.93	212,997	4,745,397	22.28
SMN-10	2,165,591	92.1	40.24	138,534	1,329,182	9.59
SMN-11	1,738,677	88.94	41.79	121,141	1,082,524	8.94
SMN-15	2,456,673	91.56	39.8	140,345	1,506,120	10.73
SMN-20	1,752,473	93.09	40.14	130,970	1,134,148	8.66
SMN-6	3,459,758	93.47	40.46	169,715	2,285,997	13.47
SMY-10	2,574,312	88.11	40.62	144,310	1,586,117	10.99
SMY-13	2,337,310	89.09	41.15	146,400	1,484,219	10.14
SMY-15	2,209,478	90.38	42.18	167,611	1,402,037	8.36
SMY-19	2,990,503	91.07	40.51	157,004	1,822,770	11.61
SMY-20	2,577,678	93.43	40.72	162,638	1,694,979	10.42
SNT-10	2,010,974	91.33	39.2	126,376	1,263,852	10
SNT-11	1,895,498	89.64	41.75	143,209	1,190,995	8.32
SNT-9	1,522,653	90.26	41.34	128,966	1,007,143	7.81
SQS-2	2,601,129	93.34	39.18	151,820	1,732,692	11.41
SQS-4	2,604,587	93.07	39.73	172,811	1,514,223	8.76
SQS-6	2,321,649	93.01	40.33	146,588	1,543,511	10.53
STW-2	3,410,534	93.91	39.24	197,803	2,256,146	11.41
STW-32	1,630,031	89.36	41.03	122,556	1,012,451	8.26
STW-6	1,683,557	90.16	41.58	133,303	1,048,042	7.86
SWN-1	1,443,782	89.74	41.73	123,312	905,586	7.34
SWN-10	1,607,077	89.75	41.5	129,435	1,016,618	7.85

SWN-15	1,736,884	89.82	41.11	136,076	1,112,047	8.17
SWN-17	1,555,196	90.34	41.11	137,402	1,007,577	7.33
SWN-20	1,694,567	92.77	41.07	130,938	1,077,689	8.23
SYL-10	1,265,699	90.48	41.53	108,177	822,279	7.6
SYL-13	1,372,180	89.45	42.03	115,837	866,876	7.48
SYL-16	1,437,051	89.98	41.59	121,044	912,443	7.54
SYL-19	2,049,128	91.1	39.04	113,496	1,257,415	11.08
SYL-20	1,650,371	88.15	40.42	103,775	998,281	9.62
TY-14	2,168,817	92.34	41.84	167,388	1,365,048	8.16
TY-23	2,095,739	91.87	39.76	130,203	1,193,826	9.17
TY-7	2,011,255	92.81	40.88	143,044	1,336,224	9.34
WC-3	1,903,158	93.32	40.89	136,330	1,267,472	9.3
WC-5	2,553,403	93.21	41.19	147,587	1,708,694	11.58
WC-6	3,143,699	92.61	41.11	153,356	2,087,843	13.61
WD-2	2,640,805	93.13	40.01	166,346	1,768,802	10.63
WD-4	2,288,701	92.77	40.27	158,446	1,495,521	9.44
WD-5	2,410,478	92.5	40.7	141,089	1,604,235	11.37
WN-1	2,883,163	92.76	40.8	182,291	1,855,321	10.18
WN-10	1,352,924	89.35	41.5	120,974	850,564	7.03
WN-2	1,908,238	90.48	41.16	138,222	1,254,135	9.07
WN-20	2,071,795	93.05	40.24	146,127	1,349,598	9.24
WN-9	2,041,075	92.55	42.43	120,636	1,344,485	11.15
XJ-12	2,456,757	89.72	39.35	128,109	1,601,871	12.5
XJ-7	1,816,165	93.07	39.59	140,338	1,180,850	8.41
XJ-9	3,583,823	89.77	40.3	154,362	2,308,898	14.96
YB-11	2,520,771	93.31	40.94	160,635	1,662,547	10.35
YB-15	2,392,955	92.91	39.87	157,849	1,549,860	9.82
YB-2	2,336,871	93.4	41.97	160,574	1,527,346	9.51
YB-4	1,731,734	90.42	41.47	134,875	1,132,513	8.4

YB-6	2,659,513	92.72	40.78	165,392	1,728,592	10.45
YC-10	2,856,865	90.91	40	143,442	1,757,867	12.25
YC-19	1,737,487	93.25	39.81	136,968	1,166,986	8.52
YC-9	2,767,271	90.35	41.76	180,699	1,798,672	9.95
YL-14	1,951,410	92.92	39.96	135,835	1,266,673	9.33
YL-16	2,439,856	90.03	42.03	147,933	1,565,877	10.59
YL-18	2,958,517	90.16	41.83	158,242	1,924,376	12.16
YNL-2	2,442,736	91.63	39.42	143,737	1,537,181	10.69
YNL-3	2,466,099	92.73	40.9	154,326	1,622,780	10.52
YNL-7	2,184,472	92.99	40.73	147,790	1,440,729	9.75
YP-11	2,348,315	92.86	40.05	169,574	1,477,574	8.71
YP-15	2,779,407	92.89	41.33	165,408	1,846,862	11.17
YP-20	2,092,280	93.09	41.13	153,938	1,393,791	9.05
YY-18	2,376,998	88.15	40.82	121,682	1,454,689	11.95
YY-2	1,829,653	87.71	39.13	113,925	1,059,770	9.3
YY-20	2,023,802	87.99	41.65	131,983	1,228,036	9.3
ZJJ-13	2,358,660	90.71	40.19	142,418	1,385,785	9.73
ZJJ-14	1,581,634	88.14	40.11	108,355	966,743	8.92
ZJJ-9	2,469,422	89.58	40.56	134,856	1,616,776	11.99
ZJK-1	1,735,800	92.44	40.79	139,614	1,101,350	7.89
ZJK-2	1,671,635	92.17	40.35	128,808	1,062,322	8.25
ZJK-3	1,752,757	92.9	39.69	133,902	1,112,059	8.31
ZJK-5	2,174,211	92.57	40.51	129,499	1,418,825	10.96
ZJK-6	2,213,874	93.17	40.09	140,851	1,462,193	10.38
ZT-11	4,394,592	93.65	41.77	245,587	2,858,046	11.64
ZT-16	2,869,446	90.39	40.7	165,612	1,896,852	11.45
ZT-20	2,303,393	88.47	40.3	134,290	1,414,086	10.53
ZX-11	2,239,597	88.96	39.47	130,033	1,401,085	10.77
ZX-5	2,148,464	90.1	39.91	132,546	1,409,315	10.63

ZX-9	2,583,901	89.58	39.74	134,352	1,662,350	12.37
ZZ-4	2,743,999	89.04	39.54	137,963	1,763,422	12.78
ZZ-6	1,730,264	91.99	40.71	134,430	1,096,808	8.16
ZZ-7	2,345,921	88.6	39.86	130,718	1,479,131	11.32

Table S2. Niche similarity detection of seven species under a single climatic variable (bio_1)

	Q.aquifolioides	*Q.guyavifolia*	*Q.monimotricha*	*Q.rehderiana*	*Q.semecarpifolia*	*Q.senescens*	*Q.spinosa*
Q.aquifolioides	NA	0.754497455	0.795253684	0.777691069	0.782483796	0.744956218	0.665707566
Q.guyavifolia	0.938786191	NA	0.833419949	0.847458729	0.846695567	0.861890571	0.86247027
Q.monimotricha	0.958000314	0.974872412	NA	0.850797681	0.852847418	0.857596207	0.765608409
Q.rehderiana	0.961615185	0.978412547	0.976703491	NA	0.831553734	0.908230464	0.826062455
Q.semecarpifolia	0.948382707	0.961626394	0.970917578	0.96787885	NA	0.822581261	0.771001058
Q.senescens	0.947872346	0.982536125	0.981915929	0.988971951	0.96794634	NA	0.830304353
Q.spinosa	0.89329485	0.977421846	0.944857269	0.965650396	0.926282159	0.973075703	NA

* The upper right and lower left corners of the table represent the ecological niche similarity between species as calculated by Schoener’s D and Hellinger distance values, respectively.

Table S3. Niche similarity detection of seven species under a single climatic variable (bio_2)

	Q.aquifolioides	*Q.guyavifolia*	*Q.monimotricha*	*Q.rehderiana*	*Q.semecarpifolia*	*Q.senescens*	*Q.spinosa*
Q.aquifolioides	NA	0.737089	0.751318	0.671995	0.775965	0.669016	0.659539
Q.guyavifolia	0.948669	NA	0.852266	0.805526	0.735844	0.807821	0.733765
Q.monimotricha	0.954106	0.96992	NA	0.808419	0.78408	0.827557	0.776537
Q.rehderiana	0.903662	0.958158	0.962157	NA	0.716756	0.800111	0.719564
Q.semecarpifolia	0.956136	0.947323	0.953859	0.929645	NA	0.789732	0.780161
Q.senescens	0.902637	0.956489	0.960834	0.972846	0.953638	NA	0.840402
Q.spinosa	0.919715	0.935139	0.957423	0.941653	0.963329	0.968422	NA

* The upper right and lower left corners of the table represent the ecological niche similarity between species as calculated by Schoener’s D and Hellinger distance values, respectively.

Table S4. Niche similarity detection of seven species under a single climatic variable (bio_3)

	Q.aquifolioides	*Q.guyavifolia*	*Q.monimotricha*	*Q.rehderiana*	*Q.semecarpifolia*	*Q.senescens*	*Q.spinosa*
Q.aquifolioides	NA	0.805301045	0.824985499	0.833202192	0.78353386	0.76463658	0.620053391
Q.guyavifolia	0.958100562	NA	0.842142635	0.830318178	0.756672447	0.824204733	0.65714798
Q.monimotricha	0.970806205	0.966319789	NA	0.865701711	0.813574004	0.855947627	0.639874278
Q.rehderiana	0.971695383	0.955981682	0.979725432	NA	0.811262276	0.854179426	0.656139589
Q.semecarpifolia	0.964612739	0.946149807	0.959336236	0.962651218	NA	0.81727996	0.735229447
Q.senescens	0.960604443	0.958843771	0.976934101	0.977071707	0.972477995	NA	0.704880078
Q.spinosa	0.897427864	0.924252517	0.909028557	0.915779287	0.953081341	0.943013267	NA

* The upper right and lower left corners of the table represent the ecological niche similarity between species as calculated by Schoener's D and Hellinger distance values, respectively.

Table S5. Niche similarity detection of seven species under a single climatic variable (bio_4)

	Q.aquifolioides	*Q.guyavifolia*	*Q.monimotricha*	*Q.rehderiana*	*Q.semecarpifolia*	*Q.senescens*	*Q.spinosa*
Q.aquifolioides	NA	0.649557	0.737996	0.69933	0.725438	0.694223	0.543384
Q.guyavifolia	0.885888	NA	0.749504	0.807435	0.68178	0.813233	0.587341
Q.monimotricha	0.919608	0.920818	NA	0.763306	0.735848	0.765	0.531542
Q.rehderiana	0.910507	0.943561	0.929859	NA	0.730998	0.850658	0.593792
Q.semecarpifolia	0.927332	0.897926	0.917748	0.931334	NA	0.748405	0.539349
Q.senescens	0.910705	0.939974	0.922235	0.963836	0.942496	NA	0.637499
Q.spinosa	0.780138	0.797142	0.758096	0.804168	0.793721	0.864483	NA

* The upper right and lower left corners of the table represent the ecological niche similarity between species as calculated by Schoener's D and Hellinger distance values, respectively.

Table S6. Niche similarity detection of seven species under a single climatic variable (bio_5)

	Q.aquifolioides	*Q.guyavifolia*	*Q.monimotricha*	*Q.rehderiana*	*Q.semecarpifolia*	*Q.senescens*	*Q.spinosa*
Q.aquifolioides	NA	0.769722	0.845766	0.798224	0.797372	0.780806	0.580568
Q.guyavifolia	0.949665	NA	0.84704	0.865142	0.892397	0.815595	0.706457
Q.monimotricha	0.973716	0.97709	NA	0.857029	0.841531	0.837878	0.655014
Q.rehderiana	0.962394	0.985021	0.976985	NA	0.856556	0.854717	0.675958
Q.semecarpifolia	0.952354	0.990183	0.970098	0.982575	NA	0.870841	0.698366
Q.senescens	0.950697	0.976493	0.968891	0.978938	0.98543	NA	0.717559
Q.spinosa	0.826968	0.90459	0.883151	0.873579	0.911176	0.916219	NA

* The upper right and lower left corners of the table represent the ecological niche similarity between species as calculated by Schoener's D and Hellinger distance values, respectively.

Table S7. Niche similarity detection of seven species under a single climatic variable (bio_6)

	Q.aquifolioides	*Q.guyavifolia*	*Q.monimotricha*	*Q.rehderiana*	*Q.semecarpifolia*	*Q.senescens*	*Q.spinosa*
Q.aquifolioides	NA	0.744986	0.778669	0.78626	0.792585	0.715059	0.681432
Q.guyavifolia	0.920231	NA	0.800737	0.868325	0.88471	0.850094	0.802322
Q.monimotricha	0.954027	0.957778	NA	0.834488	0.853098	0.848485	0.800978
Q.rehderiana	0.945444	0.979952	0.965532	NA	0.882503	0.896177	0.8179
Q.semecarpifolia	0.949041	0.97262	0.969762	0.98152	NA	0.839848	0.792941
Q.senescens	0.923028	0.978647	0.97296	0.988158	0.972374	NA	0.831438
Q.spinosa	0.894429	0.964299	0.95023	0.965502	0.944485	0.977554	NA

* The upper right and lower left corners of the table represent the ecological niche similarity between species as calculated by Schoener's D and Hellinger distance values, respectively.

Table S8. Niche similarity detection of seven species under a single climatic variable (bio_7)

	Q.aquifolioides	*Q.guyavifolia*	*Q.monimotricha*	*Q.rehderiana*	*Q.semecarpifolia*	*Q.senescens*	*Q.spinosa*
Q.aquifolioides	NA	0.713089	0.722965	0.672163	0.744923	0.671098	0.818692
Q.guyavifolia	0.922629	NA	0.854499	0.891452	0.813276	0.896346	0.678927
Q.monimotricha	0.938902	0.973867	NA	0.826252	0.81631	0.833098	0.668033
Q.rehderiana	0.891363	0.976556	0.956039	NA	0.817073	0.914631	0.644535
Q.semecarpifolia	0.946118	0.962925	0.967924	0.959457	NA	0.792701	0.712805
Q.senescens	0.904856	0.977583	0.965197	0.990076	0.962137	NA	0.636183
Q.spinosa	0.939061	0.90557	0.89404	0.877403	0.919531	0.878707	NA

* The upper right and lower left corners of the table represent the ecological niche similarity between species as calculated by Schoener's D and Hellinger distance values, respectively.

Table S9. Niche similarity detection of seven species under a single climatic variable (bio_8)

	Q.aquifolioides	*Q.guyavifolia*	*Q.monimotricha*	*Q.rehderiana*	*Q.semecarpifolia*	*Q.senescens*	*Q.spinosa*
Q.aquifolioides	NA	0.777898	0.813343	0.798418	0.812889	0.77744	0.625651
Q.guyavifolia	0.951984	NA	0.880133	0.885859	0.911742	0.877664	0.807988
Q.monimotricha	0.965226	0.980782	NA	0.860739	0.875705	0.888482	0.714335
Q.rehderiana	0.965075	0.986314	0.9824	NA	0.849873	0.867359	0.771574
Q.semecarpifolia	0.960834	0.993139	0.978595	0.980238	NA	0.873679	0.770624
Q.senescens	0.954708	0.983051	0.975792	0.975522	0.979024	NA	0.791016
Q.spinosa	0.878116	0.96331	0.922579	0.938841	0.949564	0.966677	NA

* The upper right and lower left corners of the table represent the ecological niche similarity between species as calculated by Schoener's D and Hellinger distance values, respectively.

Table S10. Niche similarity detection of seven species under a single climatic variable (bio_9)

	Q.aquifolioides	*Q.guyavifolia*	*Q.monimotricha*	*Q.rehderiana*	*Q.semecarpifolia*	*Q.senescens*	*Q.spinosa*
Q.aquifolioides	NA	0.758243	0.774257	0.772014	0.735009	0.722146	0.694924
Q.guyavifolia	0.930313	NA	0.814428	0.869384	0.765397	0.830445	0.804529
Q.monimotricha	0.957242	0.967258	NA	0.842307	0.839442	0.870108	0.79844
Q.rehderiana	0.945887	0.977396	0.972305	NA	0.786318	0.886416	0.806915
Q.semecarpifolia	0.932352	0.922675	0.96239	0.943055	NA	0.832956	0.720817
Q.senescens	0.933517	0.974982	0.981425	0.985243	0.960987	NA	0.781718
Q.spinosa	0.896867	0.964123	0.951218	0.962949	0.905578	0.958473	NA

* The upper right and lower left corners of the table represent the ecological niche similarity between species as calculated by Schoener’s D and Hellinger distance values, respectively.

Table S11. Niche similarity detection of seven species under a single climatic variable (bio_10)

	Q.aquifolioides	*Q.guyavifolia*	*Q.monimotricha*	*Q.rehderiana*	*Q.semecarpifolia*	*Q.senescens*	*Q.spinosa*
Q.aquifolioides	NA	0.766465	0.814062	0.801729	0.80373	0.778854	0.590543
Q.guyavifolia	0.950033	NA	0.882483	0.890956	0.896389	0.854433	0.730452
Q.monimotricha	0.963177	0.983939	NA	0.871456	0.857303	0.871343	0.662675
Q.rehderiana	0.964889	0.987707	0.98439	NA	0.836367	0.852842	0.692592
Q.semecarpifolia	0.957395	0.990896	0.97529	0.977103	NA	0.864852	0.734197
Q.senescens	0.951826	0.976599	0.971368	0.971359	0.979581	NA	0.757572
Q.spinosa	0.835256	0.896544	0.86836	0.875315	0.898021	0.938271	NA

* The upper right and lower left corners of the table represent the ecological niche similarity between species as calculated by Schoener’s D and Hellinger distance values, respectively.

Table S12. Niche similarity detection of seven species under a single climatic variable (bio_11)

	Q.aquifolioides	*Q.guyavifolia*	*Q.monimotricha*	*Q.rehderiana*	*Q.semecarpifolia*	*Q.senescens*	*Q.spinosa*
Q.aquifolioides	NA	0.759251	0.780965	0.779928	0.748274	0.735782	0.708635
Q.guyavifolia	0.932188	NA	0.830617	0.876511	0.783037	0.843082	0.804363
Q.monimotricha	0.957916	0.970019	NA	0.850501	0.859966	0.876377	0.812593
Q.rehderiana	0.953839	0.978077	0.97268	NA	0.79457	0.888414	0.80814
Q.semecarpifolia	0.937785	0.930667	0.969289	0.947963	NA	0.848585	0.732843
Q.senescens	0.93767	0.978874	0.984394	0.986277	0.965841	NA	0.792677
Q.spinosa	0.908439	0.968553	0.959306	0.96605	0.918222	0.966213	NA

* The upper right and lower left corners of the table represent the ecological niche similarity between species as calculated by Schoener's D and Hellinger distance values, respectively.

Table S13. Niche similarity detection of seven species under a single climatic variable (bio_12)

	Q.aquifolioides	*Q.guyavifolia*	*Q.monimotricha*	*Q.rehderiana*	*Q.semecarpifolia*	*Q.senescens*	*Q.spinosa*
Q.aquifolioides	NA	0.72105	0.70306	0.691922	0.760111	0.611036	0.668416
Q.guyavifolia	0.910395	NA	0.853867	0.786124	0.741269	0.744426	0.688609
Q.monimotricha	0.882772	0.961176	NA	0.842363	0.768874	0.811252	0.68657
Q.rehderiana	0.863123	0.949251	0.962391	NA	0.79359	0.821452	0.743328
Q.semecarpifolia	0.931455	0.93772	0.943642	0.942597	NA	0.713841	0.745005
Q.senescens	0.864695	0.936398	0.960518	0.97036	0.933811	NA	0.599857
Q.spinosa	0.86187	0.920583	0.901294	0.948703	0.929908	0.888112	NA

* The upper right and lower left corners of the table represent the ecological niche similarity between species as calculated by Schoener's D and Hellinger distance values, respectively.

Table S14. Niche similarity detection of seven species under a single climatic variable (bio_13)

	Q.aquifolioides	*Q.guyavifolia*	*Q.monimotricha*	*Q.rehderiana*	*Q.semecarpifolia*	*Q.senescens*	*Q.spinosa*
Q.aquifolioides	NA	0.704454	0.687455	0.668474	0.75586	0.636994	0.840306
Q.guyavifolia	0.921129	NA	0.875598	0.849304	0.824866	0.850874	0.747175
Q.monimotricha	0.894938	0.977082	NA	0.873445	0.834191	0.867829	0.744145
Q.rehderiana	0.86933	0.961644	0.975097	NA	0.83429	0.84697	0.748225
Q.semecarpifolia	0.941813	0.971266	0.971466	0.955975	NA	0.787057	0.814849
Q.senescens	0.880711	0.972128	0.980355	0.977852	0.954464	NA	0.688733
Q.spinosa	0.954168	0.948109	0.930247	0.926201	0.96567	0.917529	NA

* The upper right and lower left corners of the table represent the ecological niche similarity between species as calculated by Schoener's D and Hellinger distance values, respectively.

Table S15. Niche similarity detection of seven species under a single climatic variable (bio_14)

	Q.aquifolioides	*Q.guyavifolia*	*Q.monimotricha*	*Q.rehderiana*	*Q.semecarpifolia*	*Q.senescens*	*Q.spinosa*
Q.aquifolioides	NA	0.775848	0.727226	0.718308	0.808478	0.649683	0.713483
Q.guyavifolia	0.963848	NA	0.853914	0.849697	0.825757	0.777356	0.729579
Q.monimotricha	0.929245	0.976396	NA	0.817483	0.760158	0.741231	0.747915
Q.rehderiana	0.907107	0.962414	0.975986	NA	0.830005	0.894724	0.764351
Q.semecarpifolia	0.961371	0.96245	0.952478	0.965526	NA	0.785732	0.797576
Q.senescens	0.888085	0.940459	0.952931	0.983238	0.963134	NA	0.731001
Q.spinosa	0.861556	0.900865	0.902216	0.915665	0.941419	0.929785	NA

* The upper right and lower left corners of the table represent the ecological niche similarity between species as calculated by Schoener’s D and Hellinger distance values, respectively.

Table S16. Niche similarity detection of seven species under a single climatic variable (bio_15)

	Q.aquifolioides	*Q.guyavifolia*	*Q.monimotricha*	*Q.rehderiana*	*Q.semecarpifolia*	*Q.senescens*	*Q.spinosa*
Q.aquifolioides	NA	0.813637	0.801667	0.790215	0.81721	0.736399	0.610245
Q.guyavifolia	0.946342	NA	0.869574	0.83345	0.806255	0.75296	0.629259
Q.monimotricha	0.954651	0.981321	NA	0.905516	0.842286	0.819403	0.657316
Q.rehderiana	0.95206	0.970112	0.984869	NA	0.890709	0.84704	0.699859
Q.semecarpifolia	0.960864	0.951528	0.967801	0.984028	NA	0.850436	0.687293
Q.senescens	0.926284	0.933575	0.955765	0.976021	0.98157	NA	0.788595
Q.spinosa	0.83075	0.842586	0.871319	0.892968	0.912668	0.948722	NA

* The upper right and lower left corners of the table represent the ecological niche similarity between species as calculated by Schoener's D and Hellinger distance values, respectively.

Table S17. Niche similarity detection of seven species under a single climatic variable (bio_16)

	Q.aquifolioides	*Q.guyavifolia*	*Q.monimotricha*	*Q.rehderiana*	*Q.semecarpifolia*	*Q.senescens*	*Q.spinosa*
Q.aquifolioides	NA	0.714105	0.687733	0.678395	0.750152	0.646384	0.761859
Q.guyavifolia	0.918544	NA	0.850159	0.827406	0.801171	0.826655	0.716085
Q.monimotricha	0.882831	0.96662	NA	0.86833	0.824124	0.885704	0.689182
Q.rehderiana	0.862983	0.95526	0.9712	NA	0.821638	0.864643	0.712298
Q.semecarpifolia	0.933498	0.960126	0.961274	0.950002	NA	0.782079	0.778437
Q.senescens	0.878779	0.961426	0.979652	0.977692	0.952544	NA	0.655952
Q.spinosa	0.933469	0.93745	0.91155	0.921329	0.95421	0.90345	NA

* The upper right and lower left corners of the table represent the ecological niche similarity between species as calculated by Schoener's D and Hellinger distance values, respectively.

Table S18. Niche similarity detection of seven species under a single climatic variable (bio_17)

	Q.aquifolioides	*Q.guyavifolia*	*Q.monimotricha*	*Q.rehderiana*	*Q.semecarpifolia*	*Q.senescens*	*Q.spinosa*
Q.aquifolioides	NA	0.781964	0.722954	0.704564	0.800275	0.648273	0.689276
Q.guyavifolia	0.955457	NA	0.878024	0.802136	0.835674	0.75518	0.731684
Q.monimotricha	0.914374	0.978434	NA	0.810497	0.765342	0.735712	0.718197
Q.rehderiana	0.889416	0.961663	0.969825	NA	0.83274	0.898434	0.728933
Q.semecarpifolia	0.95501	0.975249	0.951413	0.963464	NA	0.788378	0.763853
Q.senescens	0.87281	0.953304	0.942152	0.981001	0.956525	NA	0.715566
Q.spinosa	0.840949	0.915835	0.8981	0.9079	0.930903	0.929613	NA

* The upper right and lower left corners of the table represent the ecological niche similarity between species as calculated by Schoener’s D and Hellinger distance values, respectively.

Table S19. Niche similarity detection of seven species under a single climatic variable (bio_18)

	Q.aquifolioides	*Q.guyavifolia*	*Q.monimotricha*	*Q.rehderiana*	*Q.semecarpifolia*	*Q.senescens*	*Q.spinosa*
Q.aquifolioides	NA	0.709545	0.679195	0.677088	0.742512	0.649215	0.823305
Q.guyavifolia	0.915667	NA	0.84161	0.817362	0.794748	0.81668	0.755684
Q.monimotricha	0.880414	0.966742	NA	0.860265	0.812456	0.881787	0.685328
Q.rehderiana	0.862289	0.950233	0.967391	NA	0.826004	0.86488	0.689974
Q.semecarpifolia	0.930323	0.958031	0.960703	0.949475	NA	0.798238	0.751495
Q.senescens	0.880472	0.961375	0.978985	0.977834	0.957033	NA	0.665084
Q.spinosa	0.955386	0.944356	0.898133	0.903068	0.941729	0.898349	NA

* The upper right and lower left corners of the table represent the ecological niche similarity between species as calculated by Schoener's D and Hellinger distance values, respectively.

Table S20. Niche similarity detection of seven species under a single climatic variable (bio_19)

	Q.aquifolioides	*Q.guyavifolia*	*Q.monimotricha*	*Q.rehderiana*	*Q.semecarpifolia*	*Q.senescens*	*Q.spinosa*
Q.aquifolioides	NA	0.784825	0.731398	0.707227	0.806873	0.648449	0.68892
Q.guyavifolia	0.949475	NA	0.866343	0.775414	0.798035	0.739998	0.728438
Q.monimotricha	0.913864	0.970577	NA	0.809382	0.76119	0.735982	0.709139
Q.rehderiana	0.887563	0.949084	0.965321	NA	0.840147	0.904269	0.724872
Q.semecarpifolia	0.955624	0.96119	0.948419	0.966147	NA	0.792061	0.74275
Q.senescens	0.872021	0.943302	0.939903	0.984476	0.955757	NA	0.71647
Q.spinosa	0.846248	0.923401	0.895663	0.91664	0.921603	0.936395	NA

* The upper right and lower left corners of the table represent the ecological niche similarity between species as calculated by Schoener’s D and Hellinger distance values, respectively.

Table S21. Detailed information on the 76 population sampling sites

Sample ID	Species	Population	Location	Latitude (°N)	Longitude (°E)	Elevation (m)	Herbarium information
QL-19	*Quercus spinosa*	QL	Taibai, Shaanxi, China	32.74	107.52	1700	KL-RBBWC
QL-13							KL-RBBWC
QL-20							KL-RBBWC
SGY-13	*Quercus spinosa*	SGY	Guangyuan, Sichuan, China	32.24	105.38	735	KL-RBBWC
SGY-18							KL-RBBWC
SGY-15							KL-RBBWC
CY-14	*Quercus spinosa*	CY	Zayu, Tibet, China	30.05	95.04	2066	KL-RBBWC
CY-19							KL-RBBWC
CY-7							KL-RBBWC
CY-9							KL-RBBWC
CY-8							KL-RBBWC
SML-1	*Quercus spinosa*	SML	Muli, Sichuan, China	27.56	101.16	2088	KL-RBBWC
SML-2							KL-RBBWC
SML-3							KL-RBBWC
SML-6							KL-RBBWC
SML-8							KL-RBBWC
SLJ-17	*Quercus spinosa*	SLJ	Lijiang, Yunnan, China	26.54	100.15	2658	KL-RBBWC
SLJ-18							KL-RBBWC
SLJ-1							KL-RBBWC
SLJ-11							KL-RBBWC
SLJ-15							KL-RBBWC
LB-13	*Quercus spinosa*	LB	Liuba, Shaanxi, China	33.28	107.09	1740	KL-RBBWC
LB-14							KL-RBBWC
LB-11							KL-RBBWC
XJ-7	*Quercus spinosa*	XJ	Xianju, Zhejiang, China	28.62	120.51	1752	KL-RBBWC
XJ-9							KL-RBBWC

XJ-12							KL-RBBWC
SQS-2	*Quercus spinosa*	SQS	Shangrao, jiangxi, China	28.93	118.08	1665	KL-RBBWC
SQS-4							KL-RBBWC
SQS-6							KL-RBBWC
JS-18	*Quercus spinosa*	JS	Jianshi, Hubei, China	30.24	110.05	*	KL-RBBWC
JS-15							KL-RBBWC
JS-4							KL-RBBWC
ZX-5	*Quercus spinosa*	ZX	Zhuxi, Hubei, China	32.26	109.64	*	KL-RBBWC
ZX-9							KL-RBBWC
ZX-11							KL-RBBWC
SNT-11	*Quercus spinosa*	SNT	Shennongjia, Hubei, China	31.57	110.47	1780	KL-RBBWC
SNT-9							KL-RBBWC
SNT-10							KL-RBBWC
YC-10	*Quercus spinosa*	YC	Yichang, Hubei, China	31.03	110.97	*	KL-RBBWC
YC-19							KL-RBBWC
YC-9							KL-RBBWC
ZZ-4	*Quercus spinosa*	ZZ	Zhouzhi, Shaanxi, China	33.82	108.01	*	KL-RBBWC
ZZ-6							KL-RBBWC
ZZ-7							KL-RBBWC
CJ-10	*Quercus spinosa*	CJ	Zhashui, Shaanxi, China	33.81	109	*	KL-RBBWC
CJ-4							KL-RBBWC
CJ-7							KL-RBBWC
NGS-13	*Quercus spinosa*	NGS	Ankang, Shaanxi, China	33.43	109.05	*	KL-RBBWC
NGS-6							KL-RBBWC
NGS-7							KL-RBBWC
ZJJ-13	*Quercus spinosa*	ZJJ	Zhangjiajie, Hunan, China	29.09	110.8	1457	KL-RBBWC
ZJJ-14							KL-RBBWC
ZJJ-9							KL-RBBWC
YL-14	*Quercus spinosa*	YL	Yuanling, Hunan, China	28.53	110.92	*	KL-RBBWC

YL-16							KL-RBBWC
YL-18							KL-RBBWC
SDL-12	*Quercus spinosa*	SDL	Dali, Yunnan, China	25.39	100.07	1960	KL-RBBWC
SDL-15							KL-RBBWC
SDL-9							KL-RBBWC
MJS-10	*Quercus spinosa*	MJS	Tianshui, Gansu, China	34.46	106.12	1330	KL-RBBWC
MJS-13							KL-RBBWC
MJS-14							KL-RBBWC
NWT-13	*Quercus spinosa*	NWT	Xi'an, Shaanxi, China	33.86	108.68	1200	KL-RBBWC
NWT-16							KL-RBBWC
NWT-18							KL-RBBWC
SL-10	*Quercus spinosa*	SL	Shangluo, Shaanxi, China	33.53	109.89	1310	KL-RBBWC
SL-17							KL-RBBWC
SL-9							KL-RBBWC
LY-16	*Quercus spinosa*	LY	Lueyang, Shaanxi, China	33.51	106.22	1680	KL-RBBWC
LY-17							KL-RBBWC
LY-5							KL-RBBWC
SHS-1	*Quercus spinosa*	SHS	Weinan, Shaanxi, China	34.53	110.1	2300	KL-RBBWC
SHS-2							KL-RBBWC
SHS-3							KL-RBBWC
DH-14	*Quercus spinosa*	DH	Dehua, Fujian, China	25.7	118.19	1789	KL-RBBWC
DH-16							KL-RBBWC
DH-8							KL-RBBWC
YB-11	*Quercus spinosa*	YB	Yanbian, Sichuan, China	27.16	101.28	2690	KL-RBBWC
YB-15							KL-RBBWC
YB-2							KL-RBBWC
YB-4							KL-RBBWC
YB-6							KL-RBBWC
SM-10	*Quercus spinosa*	SM	Mao, Sichuan, China	31.74	103.95	*	KL-RBBWC

SM-13							KL-RBBWC
SM-14							KL-RBBWC
SM-3							KL-RBBWC
SM-7							KL-RBBWC
SMN-10	*Quercus spinosa*	SMN	Mianning, Sichuan, China	28.65	102.19	2108	KL-RBBWC
SMN-11							KL-RBBWC
SMN-15							KL-RBBWC
SMN-20							KL-RBBWC
SMN-6							KL-RBBWC
KX-1	*Quercus spinosa*	KX	Kai, Chongqing, China	31.66	108.78	2289	KL-RBBWC
KX-2							KL-RBBWC
KX-3							KL-RBBWC
STW-2	*Quercus spinosa*	STW	Nantou, Taiwan, China	23.99	121.61	2410	KL-RBBWC
STW-32							KL-RBBWC
STW-6							KL-RBBWC
FYS-11	*Quercus spinosa*	FYS	Fuyuan, Yunnan, China	25.38	104.16	2333	KL-RBBWC
FYS-15							KL-RBBWC
FYS-20							KL-RBBWC
CK-11	*Quercus spinosa*	CK	Luquan, Yunnan, China	26.04	102.82	2769	KL-RBBWC
CK-16							KL-RBBWC
CK-20							KL-RBBWC
CK-5							KL-RBBWC
CK-6							KL-RBBWC
DP-2	*Quercus spinosa*	DP	Shimen, Hunan, China	29.94	110.78	1843	KL-RBBWC
DP-23							KL-RBBWC
DP-5							KL-RBBWC
JL-2	*Quercus spinosa*	JL	Jiulong, Sichuan, China	29.24	101.56	3764	KL-RBBWC
JL-4							KL-RBBWC
JL-9							KL-RBBWC

YP-11	*Quercus spinosa*	YP	Liangdang, Gansu, China	33.64	106.47	1945	KL-RBBWC
YP-15							KL-RBBWC
YP-20							KL-RBBWC
WD-2	*Quercus spinosa*	WD	Shiyan, Hubei, China	32.51	111.09	*	KL-RBBWC
WD-4							KL-RBBWC
WD-5							KL-RBBWC
TY-14	*Quercus spinosa*	TY	Zhen'an, Shaanxi, China	33.31	109.04	*	KL-RBBWC
TY-23							KL-RBBWC
TY-7							KL-RBBWC
DL-2	*Quercus spinosa*	DL	Lanping, Yunnan, China	26.41	99.41	*	KL-RBBWC
DL-5							KL-RBBWC
DL-8							KL-RBBWC
SBM-14	*Quercus spinosa*	SBM	Bomi, Tibet, China	30.16	95.22	3107	KL-RBBWC
SBM-16							KL-RBBWC
SBM-17							KL-RBBWC
SBM-19							KL-RBBWC
SBM-3							KL-RBBWC
AMX-10	*Quercus aquifolioides*	AMX	Mao, Sichuan, China	31.37	103.24	1954	KL-RBBWC
AMX-11							KL-RBBWC
AMX-16							KL-RBBWC
AMX-17							KL-RBBWC
AMX-19							KL-RBBWC
AHS-10	*Quercus aquifolioides*	AHS	Heishui, Sichuan, China	32.05	102.55	2965	KL-RBBWC
AHS-13							KL-RBBWC
AHS-15							KL-RBBWC
MAK-10	*Quercus aquifolioides*	MAK	Barkam, Sichuan, China	31.54	101.71	2770	KL-RBBWC
MAK-11							KL-RBBWC
MAK-12							KL-RBBWC
MAK-3							KL-RBBWC

MK-11	*Quercus aquifolioides*	MK	Markam, Tibet, China	29.41	98.36	4049	KL-RBBWC
MK-20							KL-RBBWC
MK-6							KL-RBBWC
ABM-14	*Quercus aquifolioides*	ABM	Bomi, Tibet, China	29.52	95.45	2728	KL-RBBWC
ABM-16							KL-RBBWC
ABM-18							KL-RBBWC
ABM-19							KL-RBBWC
ABM-9							KL-RBBWC
ALJ-11	*Quercus aquifolioides*	ALJ	Lijiang, Yunnan, China	26.53	100.14	2658	KL-RBBWC
ALJ-13							KL-RBBWC
ALJ-15							KL-RBBWC
ALJ-19							KL-RBBWC
ALJ-9							KL-RBBWC
AYB-11	*Quercus aquifolioides*	AYB	Yanbian, Sichuan, China	27.15	101.27	2691	KL-RBBWC
AYB-2							KL-RBBWC
AYB-31							KL-RBBWC
AYB-4							KL-RBBWC
AYB-9							KL-RBBWC
WC-3	*Quercus aquifolioides*	WC	Wenchuan, Sichuan, China	31.53	103.5	*	KL-RBBWC
WC-5							KL-RBBWC
WC-6							KL-RBBWC
NX-1	*Quercus aquifolioides*	NX	Shangri-La, Yunnan, China	28	99.55	2900	KL-RBBWC
NX-3							KL-RBBWC
NX-4							KL-RBBWC
YNL-2	*Quercus aquifolioides*	YNL	Ninglang, Yunnan, China	27.29	100.85	*	KL-RBBWC
YNL-3							KL-RBBWC
YNL-7							KL-RBBWC
HLG-10	*Quercus aquifolioides*	HLG	Jiulong, Sichuan, China	29	101.51	*	KL-RBBWC
HLG-11							KL-RBBWC

HLG-3							KL-RBBWC
NTC-2	*Quercus aquifolioides*	NTC	Shangri-La, Yunnan, China	27.82	99.71	*	KL-RBBWC
NTC-6							KL-RBBWC
NTC-9							KL-RBBWC
AML-10	*Quercus aquifolioides*	AML	Muli, Sichuan, China	27.75	101.22	*	KL-RBBWC
AML-2							KL-RBBWC
AML-3							KL-RBBWC
AML-6							KL-RBBWC
AML-9							KL-RBBWC
RMX-11	*Quercus rehderiana*	RMX	Mao, Sichuan, China	31.38	103.25	1954	KL-RBBWC
RMX-13							KL-RBBWC
RMX-14							KL-RBBWC
RMX-2							KL-RBBWC
RMX-6							KL-RBBWC
RMAK-12	*Quercus rehderiana*	RMAK	Barkam, Sichuan, China	31.55	101.72	2560	KL-RBBWC
RMAK-15							KL-RBBWC
RMAK-19							KL-RBBWC
RMAK-4							KL-RBBWC
RMAK-5							KL-RBBWC
RML-1	*Quercus rehderiana*	RML	Muli, Sichuan, China	27.55	101.18	2100	KL-RBBWC
RML-12							KL-RBBWC
RML-19							KL-RBBWC
RML-6							KL-RBBWC
RML-9							KL-RBBWC
RLJ-14	*Quercus rehderiana*	RLJ	Lijiang, Yunnan, China	26.56	100.15	2700	KL-RBBWC
RLJ-15							KL-RBBWC
RLJ-18							KL-RBBWC
RLJ-2							KL-RBBWC
RLJ-9							KL-RBBWC

WN-1	*Quercus rehderiana*	WN	Weining, Guizhou, China	26.46	104.12	2136	KL-RBBWC
WN-10							KL-RBBWC
WN-2							KL-RBBWC
WN-20							KL-RBBWC
WN-9							KL-RBBWC
LD-3	*Quercus rehderiana*	LD	Luding, Sichuan, China	29.54	102.12	1789	KL-RBBWC
LD-5							KL-RBBWC
LD-6							KL-RBBWC
PLJ-12	*Quercus rehderiana*	PLJ	Lijiang, Yunnan, China	26.52	100.13	2658	KL-RBBWC
PLJ-15							KL-RBBWC
PLJ-16							KL-RBBWC
PLJ-17							KL-RBBWC
PLJ-18							KL-RBBWC
MEK-12	*Quercus guyavifolia*	MEK	Barkam, Sichuan, China	31.91	102.2	*	KL-RBBWC
MEK-13							KL-RBBWC
MEK-14							KL-RBBWC
MEK-15							KL-RBBWC
MEK-16							KL-RBBWC
DQ-1	*Quercus guyavifolia*	DQ	Deqing, Yunnan, China	28.45	98.88	*	KL-RBBWC
DQ-12							KL-RBBWC
DQ-9							KL-RBBWC
GYL-12	*Quercus guyavifolia*	GYL	Lijiang, Yunnan, China	27.03	100.26	*	KL-RBBWC
GYL-16							KL-RBBWC
GYL-18							KL-RBBWC
GYL-20							KL-RBBWC
GYL-6							KL-RBBWC
GMY-11	*Quercus guyavifolia*	GMY	Yanyuan, Sichuan, China	27.57	101.36	*	KL-RBBWC
GMY-13							KL-RBBWC
GMY-14							KL-RBBWC

GMY-17							KL-RBBWC
GMY-20							KL-RBBWC
ZJK-1	*Quercus guyavifolia*	ZJK	Luquan, Yunnan, China	26.03	102.82	2861	KL-RBBWC
ZJK-2							KL-RBBWC
ZJK-3							KL-RBBWC
ZJK-5							KL-RBBWC
ZJK-6							KL-RBBWC
BM-14	*Quercus guyavifolia*	BM	Bomi, Tibet, China	29.86	95.77	*	KL-RBBWC
BM-17							KL-RBBWC
BM-19							KL-RBBWC
BM-20							KL-RBBWC
BM-9							KL-RBBWC
GML-1	*Quercus guyavifolia*	GML	Muli, Sichuan, China	27.57	101.36	*	KL-RBBWC
GML-11							KL-RBBWC
GML-20							KL-RBBWC
GML-3							KL-RBBWC
GML-5							KL-RBBWC
SCY-15	*Quercus senescens*	SCY	Zayu, Tibet, China	28.67	97.47	*	KL-RBBWC
SCY-17							KL-RBBWC
SCY-5							KL-RBBWC
SCY-6							KL-RBBWC
SCY-9							KL-RBBWC
SYL-10	*Quercus senescens*	SYL	Lijiang, Yunnan, China	27.03	100.26	*	KL-RBBWC
SYL-13							KL-RBBWC
SYL-16							KL-RBBWC
SYL-19							KL-RBBWC
SYL-20							KL-RBBWC
SMY-10	*Quercus senescens*	SMY	Muli, Sichuan, China	27.57	101.36	*	KL-RBBWC
SMY-13							KL-RBBWC

SMY-15							KL-RBBWC
SMY-19							KL-RBBWC
SMY-20							KL-RBBWC
ZT-11	*Quercus senescens*	ZT	Daguan, Yunnan, China	27.75	103.89	*	KL-RBBWC
ZT-16							KL-RBBWC
ZT-20							KL-RBBWC
SWN-1	*Quercus senescens*	SWN	Weining, Guizhou, China	26.87	104.28	*	KL-RBBWC
SWN-10							KL-RBBWC
SWN-15							KL-RBBWC
SWN-17							KL-RBBWC
SWN-20							KL-RBBWC
KM-13	*Quercus senescens*	KM	Xundian, Yunnan, China	25.67	102.85	2535	KL-RBBWC
KM-3							KL-RBBWC
KM-4							KL-RBBWC
MN-1	*Quercus senescens*	MN	Mianning, Sichuan, China	28.63	102.18	2108	KL-RBBWC
MN-11							KL-RBBWC
MN-14							KL-RBBWC
MN-5							KL-RBBWC
MN-7							KL-RBBWC
PMS-11	*Quercus monimotricha*	PMS	Kangding, Sichuan, China	30	101.97	*	KL-RBBWC
PMS-18							KL-RBBWC
PMS-5							KL-RBBWC
YY-18	*Quercus monimotricha*	YY	Yanyuan, Sichuan, China	27.43	101.51	*	KL-RBBWC
YY-2							KL-RBBWC
YY-20							KL-RBBWC
KD-12	*Quercus monimotricha*	KD	Kangding, Sichuan, China	30	101.96	*	KL-RBBWC
KD-13							KL-RBBWC
KD-3							KL-RBBWC
LZ-3	*Quercus semecarpifolia*	LZ	Linzhi, Tibet, China	29.66	94.34	*	KL-RBBWC

LZ-4	KL-RBBWC
LZ-8	KL-RBBWC
LZ-1	KL-RBBWC
LZ-6	KL-RBBWC

KL-RBBWC: Key Laboratory of Resource Biology and Biotechnology in Western China, Ministry of Education, Northwest University

Table S22. Detailed morphological differences among the seven species

Species	Leaf morphological characteristics	Leaf image	Fruit types and morphological characteristics	Fruit images
Quercus spinosa	● The leaves are obovate or elliptical, with a bluntly rounded apex and a base nearly round or shallowly cordate.<br>● The leaf margins are either entire or sparsely serrated.<br>● The lower part of the midvein on the underside of old leaves is densely covered with grayish-brown stellate hairs, while the rest is hairless.<br>● The midvein and lateral veins on the upper surface of the leaves are sunken.	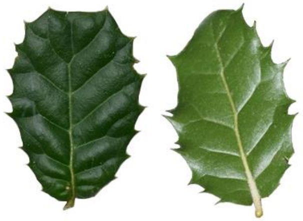	● The cupule is cup-shaped, enclosing 1/4 to 1/3 of the nut.	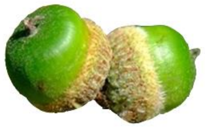
Quercus aquifolioides	● The leaves are elliptical or obovate, with the apices of old leaves being round, occasionally short-acuminate, and the bases nearly round or shallowly cordate.<br>● The leaf margins are entire, rarely serrated.<br>● The upper surface of old leaves is hairless, while the underside is covered with stellate hairs and mealy scurf, which gradually fall off.	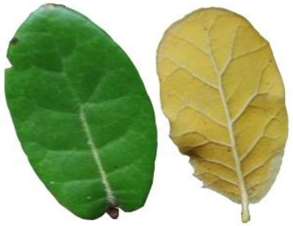	● The cupule is cup-shaped, and the fruit is ovoid, hairless.	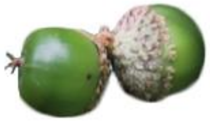

Quercus rehderiana	● The leaves are flat, elliptical or obovate-elliptical, with a bluntly rounded apex and a round base.<br>● The leaf margins are either entire or have a few spine-like teeth.<br>● The upper surface of the leaf is hairless, while the base of the midvein on the underside is densely covered with short, grayish-yellow stellate hairs.<br>● The midvein is zigzag-shaped and hairless.	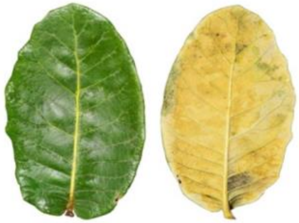	● The nuts are ovate or nearly spherical, and hairless.	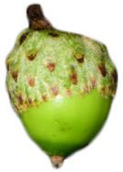
Quercus guyavifolia	● The leaves are obovate or elliptical, with a bluntly rounded or shortly blunt-tipped apex, and a base that is nearly round or shallowly cordate.<br>● The leaf margins have spine-like teeth or are nearly entire.<br>● The underside of the leaves is densely covered with reddish-brown or yellowish-brown stellate hairs, single hairs, and mealy scurf.<br>● The midvein often zigzags.	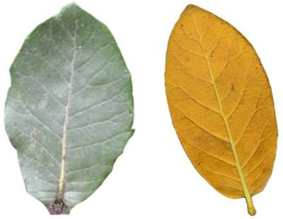	● The cupule is shallowly cup-shaped, and the bracts are narrow ovate, covered with brown villi.<br>● The fruit is ovoid-spherical.	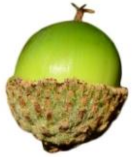

Quercus monimotricha	●The leaves are elliptical or obovate, with a bluntly rounded or shortly acute apex, and a base that is nearly round or shallowly cordate.<br>●The leaf margins have long spine-like teeth, rarely nearly entire.<br>●The upper and lower surfaces of old leaves are nearly the same color. Along the midvein of the upper surface, there are sparse stellate hairs, while the underside has dark brown stellate hairs or is nearly hairless.<br>●The petioles are densely covered with hair.	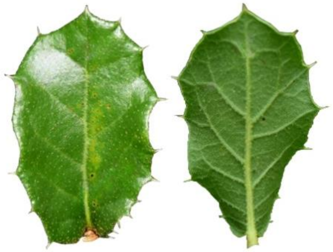	●The cupule is cup-shaped, and the bracts are covered with grayish-brown villi.<br>●The fruit is ovoid, yellowish-brown.	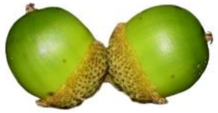
Quercus semecarpifolia	●The leaves are elliptical or oblong-elliptical, with a bluntly rounded apex and a shallowly cordate base.<br>●The leaf margins are either entire or have spine-like serrations.<br>●The upper surface of the leaves is hairless or has sparse stellate hairs, while the underside is covered with brown stellate hairs and mealy powder.<br>●The petioles are either hairless or have fine hairs.	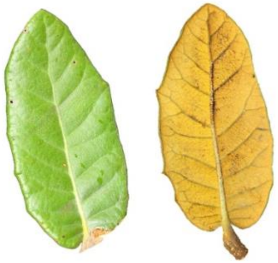	●The nuts are nearly spherical, hairless or slightly hairy near the top, and sometimes tinged with purplish-brown.<br>●The fruit umbilicus is flat or slightly raised.	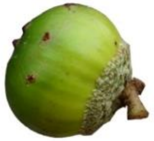

Quercus senescens	●The leaves are oblong or obovate-elliptical, with a bluntly rounded apex and a base that is nearly round or shallowly cordate.<br>●The leaf margins are either entire or have spine-like teeth.<br>●The young leaves are covered with grayish-yellow hairs on both surfaces, while the older leaves are nearly hairless on the upper surface and densely covered with grayish-brown or grayish-yellow villi on the underside.	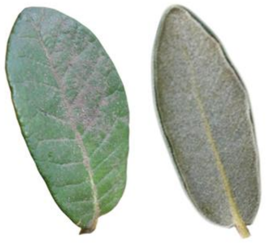	●The fruit umbilicus is raised or protuberant.	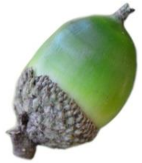

Table S23. Maximum likelihood fits of 24 different nucleotide substitution models

Model	#Param	BIC	AICc	lnL	Invariant	Gamma	R	Freq A	Freq T	Freq C	Freq G	A=>T	A=>C	A=>G	T=>A	T=>C	T=>G	C=>A	C=>T	C=>G	G=>A	G=>T	G=>C
GTR+G+I	593	1394833.76	1386888.76	-692851.308	0.00553	1.48255	2.38988	0.19353	0.18607	0.31194	0.30846	0.06	0.04	0.26	0.07	0.18	0.03	0.02	0.11	0.03	0.16	0.02	0.03
GTR+G	592	1394840.97	1386909.37	-692862.61	n/a	1.43965	2.39024	0.19353	0.18607	0.31194	0.30846	0.06	0.04	0.26	0.07	0.18	0.03	0.02	0.11	0.03	0.16	0.02	0.03
K2+G	585	1395224.31	1387386.49	-693108.174	n/a	1.47023	3.19139	0.25	0.25	0.25	0.25	0.03	0.03	0.19	0.03	0.19	0.03	0.03	0.19	0.03	0.19	0.03	0.03
K2+G+I	586	1395239.77	1387388.55	-693108.203	0.00001	1.47034	3.19135	0.25	0.25	0.25	0.25	0.03	0.03	0.19	0.03	0.19	0.03	0.03	0.19	0.03	0.19	0.03	0.03
T92+G	586	1406120.79	1398269.57	-698548.715	n/a	1.48666	2.92862	0.1898	0.1898	0.3102	0.3102	0.02	0.04	0.23	0.02	0.23	0.04	0.02	0.14	0.04	0.14	0.02	0.04
HKY+G	588	1406125.13	1398247.11	-698535.484	n/a	1.4865	2.92834	0.19353	0.18607	0.31194	0.30846	0.02	0.04	0.23	0.02	0.24	0.04	0.02	0.14	0.04	0.15	0.02	0.04
T92+G+I	587	1406127.91	1398263.3	-698544.578	0.00001	1.48657	2.93319	0.1898	0.1898	0.3102	0.3102	0.02	0.04	0.23	0.02	0.23	0.04	0.02	0.14	0.04	0.14	0.02	0.04
HKY+G+I	589	1406132.22	1398240.81	-698531.334	0	1.48632	2.93291	0.19353	0.18607	0.31194	0.30846	0.02	0.04	0.23	0.02	0.24	0.04	0.02	0.14	0.04	0.15	0.02	0.04
TN93+G	589	1406147.75	1398256.34	-698539.097	n/a	1.48666	2.92897	0.19353	0.18607	0.31194	0.30846	0.02	0.04	0.23	0.02	0.24	0.04	0.02	0.14	0.04	0.15	0.02	0.04
TN93+G+I	590	1406150.97	1398246.16	-698533.007	0.00001	1.48646	2.93501	0.19353	0.18607	0.31194	0.30846	0.02	0.04	0.23	0.02	0.24	0.04	0.02	0.14	0.04	0.15	0.02	0.04
K2+I	585	1420265.41	1412427.59	-705628.724	0.06406	n/a	3.07125	0.25	0.25	0.25	0.25	0.03	0.03	0.19	0.03	0.19	0.03	0.03	0.19	0.03	0.19	0.03	0.03
GTR+I	592	1423071.25	1415139.64	-706977.75	0.06195	n/a	2.3363	0.19353	0.18607	0.31194	0.30846	0.06	0.04	0.23	0.06	0.2	0.04	0.02	0.12	0.03	0.15	0.02	0.03
K2	584	1425053.16	1417228.74	-708030.3	n/a	n/a	3.06364	0.25	0.25	0.25	0.25	0.03	0.03	0.19	0.03	0.19	0.03	0.03	0.19	0.03	0.19	0.03	0.03
GTR	591	1429030.47	1421112.27	-709965.061	n/a	n/a	2.34404	0.19353	0.18607	0.31194	0.30846	0.06	0.04	0.23	0.06	0.2	0.04	0.02	0.12	0.03	0.15	0.02	0.03
T92+I	586	1432033.41	1424182.19	-711505.027	0.06352	n/a	2.91903	0.1898	0.1898	0.3102	0.3102	0.02	0.04	0.23	0.02	0.23	0.04	0.02	0.14	0.04	0.14	0.02	0.04
HKY+I	588	1432046.06	1424168.04	-711495.951	0.06354	n/a	2.91876	0.19353	0.18607	0.31194	0.30846	0.02	0.04	0.23	0.02	0.24	0.04	0.02	0.14	0.04	0.15	0.02	0.04
TN93+I	589	1432063.3	1424171.89	-711496.871	0.06353	n/a	2.93612	0.19353	0.18607	0.31194	0.30846	0.02	0.04	0.23	0.02	0.24	0.04	0.02	0.14	0.04	0.14	0.02	0.04
T92	585	1437588.87	1429751.05	-714290.455	n/a	n/a	2.91505	0.1898	0.1898	0.3102	0.3102	0.02	0.04	0.23	0.02	0.23	0.04	0.02	0.14	0.04	0.14	0.02	0.04
HKY	587	1437597.13	1429732.51	-714279.184	n/a	n/a	2.91478	0.19353	0.18607	0.31194	0.30846	0.02	0.04	0.23	0.02	0.24	0.04	0.02	0.14	0.04	0.15	0.02	0.04
TN93	588	1437609	1429730.99	-714277.422	n/a	n/a	2.93458	0.19353	0.18607	0.31194	0.30846	0.02	0.04	0.23	0.02	0.24	0.04	0.02	0.14	0.04	0.14	0.02	0.04
JC+G	584	1487791.83	1479967.41	-739399.633	n/a	1.64094	0.5	0.25	0.25	0.25	0.25	0.08	0.08	0.08	0.08	0.08	0.08	0.08	0.08	0.08	0.08	0.08	0.08
JC+G+I	585	1487807.23	1479969.41	-739399.634	0	1.64093	0.5	0.25	0.25	0.25	0.25	0.08	0.08	0.08	0.08	0.08	0.08	0.08	0.08	0.08	0.08	0.08	0.08
JC+I	584	1510075.9	1502251.48	-750541.668	0.06404	n/a	0.5	0.25	0.25	0.25	0.25	0.08	0.08	0.08	0.08	0.08	0.08	0.08	0.08	0.08	0.08	0.08	0.08
JC	583	1514733.22	1506922.2	-752878.029	n/a	n/a	0.5	0.25	0.25	0.25	0.25	0.08	0.08	0.08	0.08	0.08	0.08	0.08	0.08	0.08	0.08	0.08	0.08

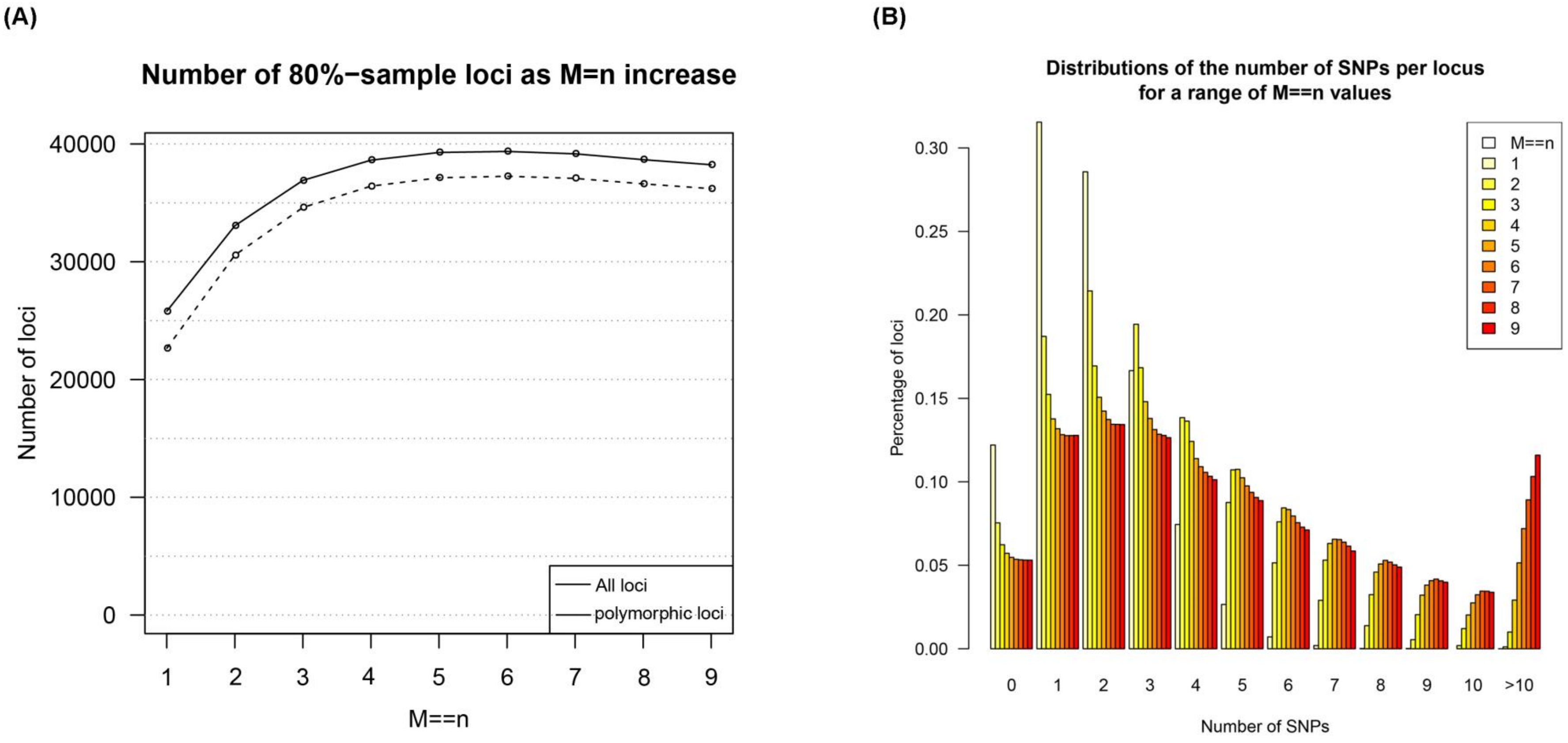

Figure S1. Parameter tuning of the test data. (A) The number of r80 sites corresponding to different *M* values. (B) Quantitative distribution of SNP sites on different loci.

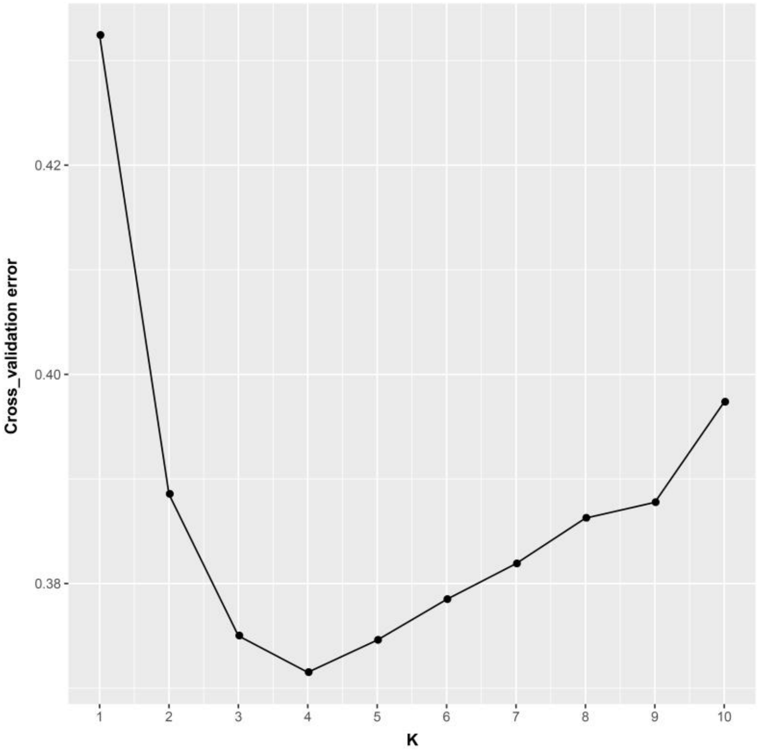

Figure S2. Cross-validation error rate results for 76 populations.

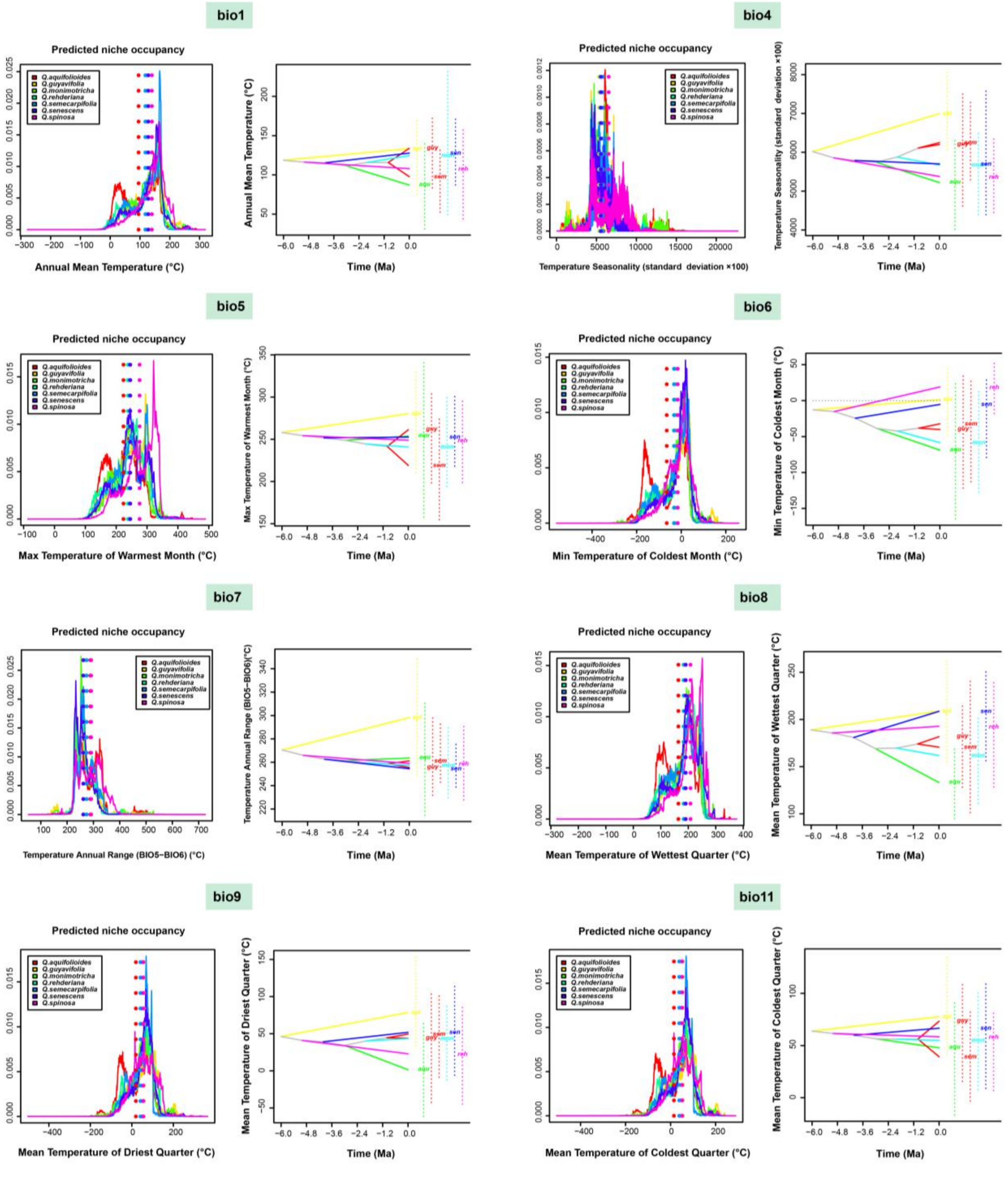

Figure S3. PNO profiles and inferred history of climatic tolerance evolution for seven species under climatic variation. The PNO horizontal axis represents the occupancy of various temperature indicators, while the vertical axis indicates the suitability of each species for each variable depicted.

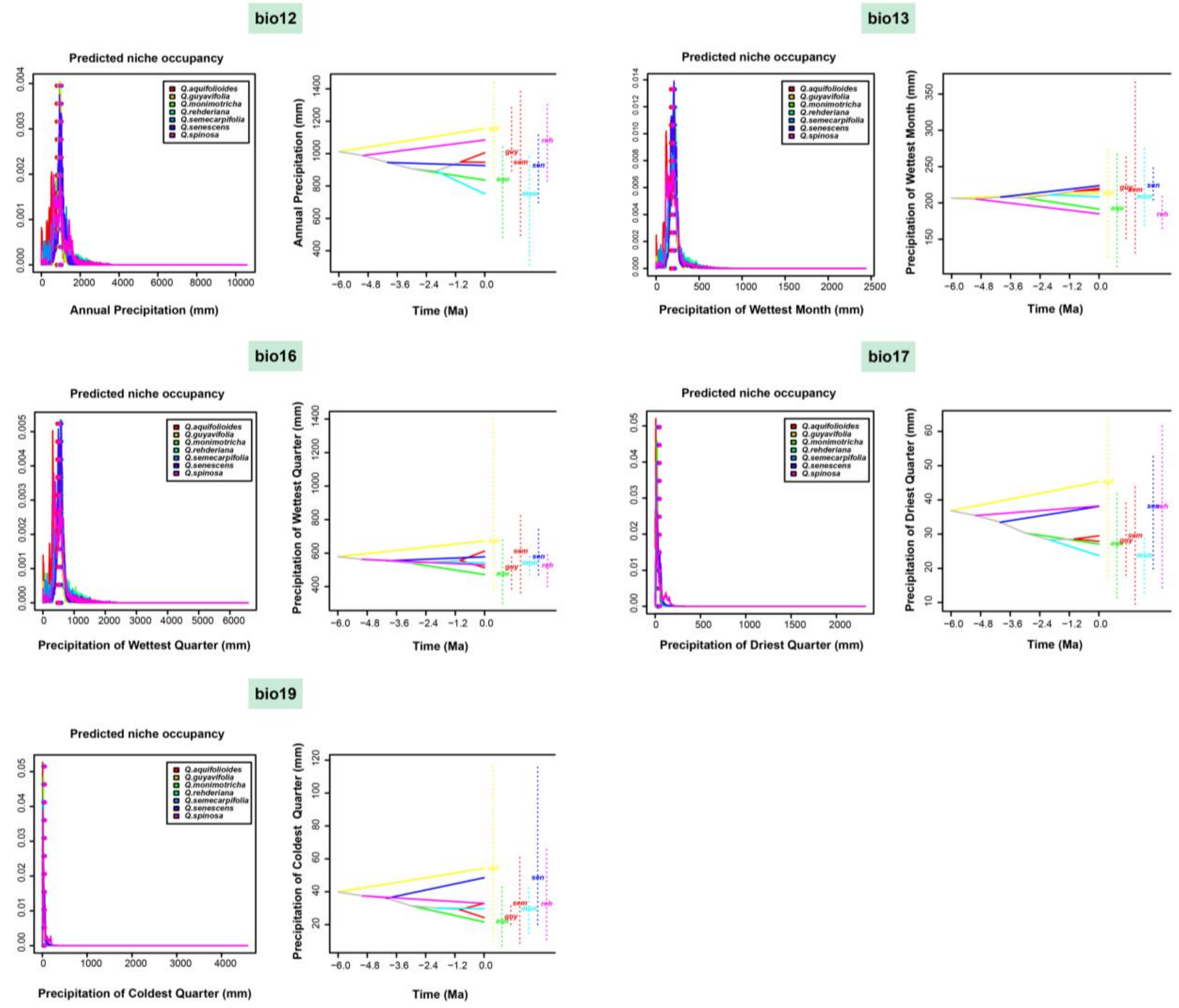

Figure S4. PNO profiles and inferred history of climatic tolerance evolution for seven species under climatic variation. The PNO horizontal axis represents the occupancy of various precipitation indicators, while the vertical axis indicates the suitability of each species for each variable depicted.
